# Strecker-Type Cyanation of Acetals Catalyzed by Tin(IV) Chloride—A Mechanistic Study

**DOI:** 10.1021/acs.joc.6c00737

**Published:** 2026-06-23

**Authors:** Ismayil M. Garazade, Armando J. L. Pombeiro, Maxim L. Kuznetsov

**Affiliations:** † Centro de Química Estrutural, Institute of Molecular Sciences, Instituto Superior Técnico, 72971 Universidade de Lisboa, Av. Rovisco Pais, Lisbon 1049-001, Portugal; ‡ Faculty of Chemistry, Baku State University, Z. Xalilov Str. 23, Baku 1148, Azerbaijan

## Abstract

The mechanism of reaction between benzaldehyde dimethyl acetal (PhCH­(OMe)_2_) and trimethylsilylcyanide (TMSCN) in the presence of SnCl_4_·5H_2_O was investigated by theoretical (DFT) and experimental (NMR) methods. Two competitive reaction channels were revealed: Strecker-type cyanation to PhCH­(OMe)­(CN) and acetal hydrolysis to benzaldehyde. The most favorable mechanism of cyanation includes coordination of TMSCN, TMS transfer to acetal, the C–O_Me_ bond rupture to give [PhCH­(OMe)]^+^ and *cis*-[SnCl_4_(NC)­(H_2_O)]^−^, and the ion recombination affording the final product. Hydrolysis includes the acetal protonation by a coordinated water molecule to form the [PhCH­(OMe)]^+^···*cis*-[SnCl_4_(OH)­(H_2_O)]^−^ ion pair, ion recombination to hemiacetal, and elimination of the second MeOH molecule to give benzaldehyde. Further evolution of benzaldehyde as well as other intermediates (i.e., PhCH­(OMe)­(NC) and HCN) is also discussed. The tin­(IV) catalyst plays a crucial role in both reaction channels. In the cyanation of acetal, the TMSCN coordination to the Sn­(IV) center activates this species toward the Si–C bond cleavage while, in the acetal hydrolysis, the highly acidic nature of water coordinated to the Sn­(IV) center facilitates the acetal protonation and the C–O bond cleavage as well as the proton transfer in the hemiacetal—the key steps in the formation of benzaldehyde.

## Graphical abstract

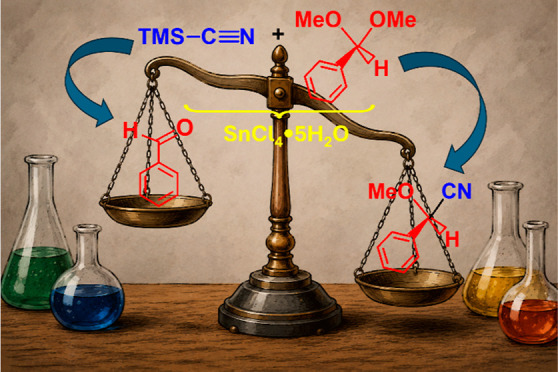

## Introduction

The Strecker and Strecker-type reactions are one of the most versatile tools toward the cyanation of various organic substrates, leading to organic nitriles.^1–7^ The original Strecker reaction comprises one-pot condensation of three components, i.e., an aldehyde, ammonia, and a cyanide group source, featuring, after subsequent hydrolysis of the organic nitrile, the corresponding α-amino acid (Scheme 1A).^8^ A popular alternative version of this reaction involves a stepwise condensation of the carbonyl compound and an amine, followed by the cyanation of the imine to give an organic nitrile (Scheme 1B). The further expansion of the reaction scope is related to the application of the cyanation substrates different from imines, such as carbonyl compounds and alkenes, which, upon treatment with a cyanide source, afford a wide variety of organic nitrile products (Scheme 1C).^9–11^

**Scheme 1:**
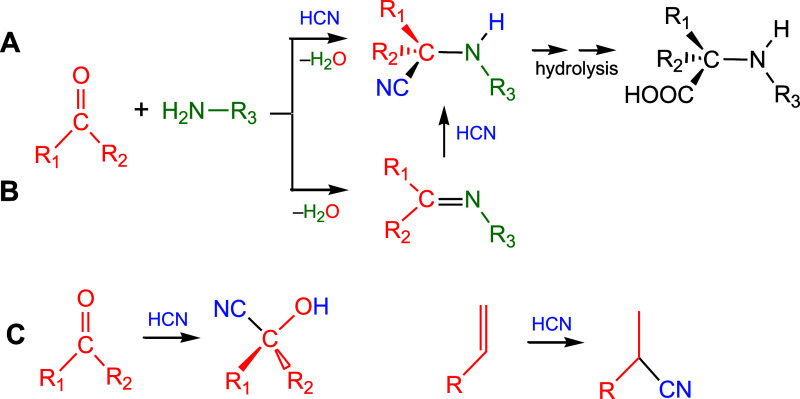
Strecker and Strecker-Type Cyanation with HCN Used as a CN Group Source

Hydrogen cyanide (HCN) is the simplest reagent applied for cyanation. However, its application is limited by high toxicity and volatility. As an alternative, trimethylsilyl cyanide (TMSCN) is commonly used in these processes as a safer and more convenient cyanation reagent.^12^ Additionally, TMSCN is typically more reactive than HCN due to the lower Si–CN bond energy compared to the H–CN one. Meanwhile, other cyanide group sources (e.g., inorganic cyanides, cyanohydrins, *N*-cyano succinimide, *N*-cyanophthalimide, *N*-cyano sulfonamides, etc.) are also known.

Besides substrates with unsaturated functional groups, acetals can also undergo cyanation in a similar Strecker-type process. However, examples of acetal cyanation are rare. They include a three-component Strecker reaction with participation of cyclopropanone hemiacetal, amines, and NaCN^13^ or acetals, amines, and TMSCN catalyzed by salts of various metals (Hf­(IV), Zr­(IV), In­(III), Fe­(III), Al­(III), Bi­(III), Sn­(II), Cu­(I)),^14^ ring-opening cyclopropanone acetal cyanation with TMSCN catalyzed by copper thiophene-2-carboxylate,^15^ ring-opening cyanation of cyclic thioacetals with phenyl thiocyanate catalyzed by rhodamine 6G in the presence of K_3_PO_4_,^16^ cyanation of *N*,*O*-acetals with TMSCN catalyzed by Ca­(NTf_2_)_2_/*n*Bu_4_NPF_6_ (NTf = trifluoromethanesulfonimide),^17^ cyanation of acetals with a sterically congested α-cyanoamine as a cyanating reagent in the presence of trichlorosilyl triflate,^18^ uncatalyzed cyanation of acetals with TMSCN under high pressure,^19^ cyanation of isochromene acetals with TMSCN catalyzed by Fe­(III) triflate,^20^ cyanation of acetals with TMSCN under acidic catalytic conditions,^21^ and that catalyzed by BiBr_3_,^22^ TiCl_4_,^23^ MgI_2_ etherate,^24^ and Cd­(II)-based coordination polymers.^25,26^

The cyanation reactions usually require the application of a catalyst to improve the yield of final products under reasonable reaction conditions and reaction time. Complexes of various metals, e.g., Al, Ti, V, Ru, Ca, Mg, Bi,^4,27,28^ Fe,^29^ Pd, Ni, Co,^30–34^ Cu,^35,36^ Zr, W,^37^ and many others, represent one of the main types of catalytic systems broadly used in these processes. Among other metals, tin is one of the least explored as a catalyst in these reactions. Several tin compounds were successfully applied as catalysts for cyanosilylation of aldehydes and ketones, i.e., terephthalate-^38^ and 3-amino-2-pyrazinecarboxylate-^39^ based Sn­(II) complexes, a Sn­(II) triflate (+)-cinchonine derivative,^40^ a functionalized stannylene cyanide,^41,42^ a Sn–W mixed oxide,^43^ a tin ion-exchanged montmorillonite catalyst,^44–46^ a tin metallocene,^47^ organotin–PTA complexes supported on mesoporous carbon,^48^ and a bimetallic Sn–Ir complex.^49^ Simple inorganic salt SnCl_2_·2H_2_O and organotin compounds Bu_2_SnO, Bu_2_SnBr_2_, and Bu_2_Sn­(OMe)_2_ revealed a catalytic activity in the synthesis of nitriles from aldehydes and oximes and in cyanotransfer from benzophenone cyanohydrin to aldehydes and imines.^50,51^ Yanagisawa et al. reported an asymmetric cyanation of β-keto esters catalyzed by a chiral tin alkoxide.^52^ Finally, a tin-exchanged zeolite and montmorillonite were applied as catalysts in the classical three-component Strecker reaction.^53,54^

Recently,^55^ we reported results of the Strecker-type cyanation of benzaldehyde dimethyl acetal PhCH­(OMe)_2_ (BDMA) with TMSCN catalyzed by several copper­(II) complexes and two tin­(IV) species, i.e., Sn­(Me)_2_Cl_2_ and SnCl_4_·5H_2_O (Scheme 2). The latter salt exhibited particularly high catalytic activity toward 2-methoxy-2-phenylacetonitrile, PhCH­(OMe)­(CN), and is very attractive for possible practical applications, being a relatively cheap, commercially available reagent.

**Scheme 2:**
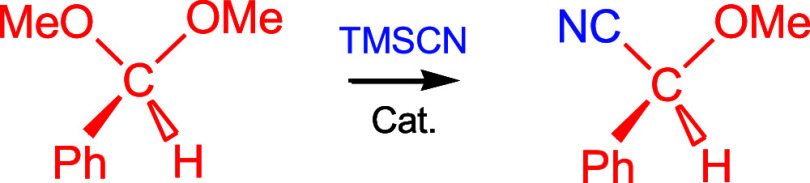
Cyanation of Benzaldehyde Dimethyl Acetal with TMSCN

One of the commonly accepted mechanisms of α-cyanosilylation of carbonyl compounds with an RCN cyanation agent includes an attack of the nitrile carbon atom at the sp^2^ carbon atom of the aldehyde or ketone with simultaneous or subsequent transfer of R to the carbonyl oxygen atom (Scheme 3)^42,56–61^ or to another part of a catalyst.^62^ The proposed role of the catalyst in these processes is an activation of either substrate or RCN or both of them by coordination to a metal center or through the formation of hydrogen bonds with the catalyst. Some alternative mechanistic schemes were also proposed.^63–68^ However, the cyanation of acetals should occur via a mechanism of a fundamentally different type since the central carbon atom of the acetal is at the sp^3^ hybridization, and the nucleophilic attack at this center is not feasible. To the best of our knowledge, no studies of the mechanism of the acetal cyanation with TMSCN have been reported.

**Scheme 3:**
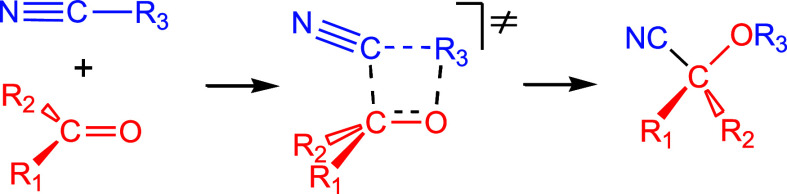
One-Step Mechanism of α-Cyanosilylation of Carbonyl Compounds

To fill this gap, we report here the results of the extensive computational study on the mechanism of the benzaldehyde dimethyl acetal PhCH­(OMe)_2_ cyanation with TMSCN, catalyzed by tin­(IV) chloride hydrate. The choice of the hydrate of SnCl_4_ instead of the anhydrous salt was based on the great performance of this catalyst found by us previously^55^ and by the fact that the tin­(IV) chloride hydrate is much more accessible, stable, easy to handle, cheap, and, therefore, more promising for practical applications compared to the anhydrous salt. The article has the following structure. First, we discuss the active form of the catalyst under experimental conditions; second, the initial activation of the acetal toward the C–OMe bond cleavage is analyzed; third, transformations of the key carbocation intermediate [PhCH­(OMe)]^+^ are revealed; fourth, evolution of the side reaction products (i.e., PhCH­(=O), PhCH­(OMe)­(NC), and HCN) is analyzed; finally, fifth, new experimental data concerning the system and reaction under study are reported and discussed to confirm the key predictions based on the theoretical calculations.

## Computational Details

Full geometry optimization of all structures and transition states (TS) was carried out at the density functional theory (DFT) level using the M06-2X functional^69^ with the help of the Gaussian-09 ^70^ program package and applying the def2-TZVP basis set and the corresponding pseudopotential describing 28 core electrons (for the Sn atom) taken from the EMSL Basis Set Exchange Library.^71^ Previously, the Minnesota family functionals demonstrated good performance in the theoretical calculations of the reaction mechanism of a deacetalization–Knoevenagel condensation reaction between benzaldehyde dimethyl acetal and malononitrile.^72^ Solvent effects were considered during the optimization, applying the polarizable continuum model in the SMD version^73^ with acetonitrile as the solvent. Cartesian d and f basis functions (6d, 10f) were used in all calculations. No symmetry operations have been applied for any of the structures calculated. The Hessian matrix was calculated analytically for the optimized structures in order to determine the location of correct minima (no imaginary frequencies) or saddle points (only one imaginary frequency) and to estimate the thermodynamic parameters, the latter being calculated at 298.15 K and 1 atm. The nature of all transition states was investigated by the analysis of vectors associated with the imaginary frequency and by the calculations of the intrinsic reaction coordinates (IRC) using the Gonzalez–Schlegel method.^74–76^

## Experimental Details

Reagents SnCl_4_·5H_2_O (≥98%), TMSCN (≥98%), BDMA (98%), and benzaldehyde (BA) (≥98%) were used as received from Thermo Scientific and Acros Organics. All reactions were conducted under standard thermal conditions in sealed glass reaction tubes. All reactions were conducted under standard thermal conditions using capped glass reaction tubes. For each experiment, the required stoichiometric amounts of reagents were used (see the Experimental Verification section). In catalytic runs, 1 mol % of SnCl_4_·5H_2_O (3.5 mg) was introduced. After the addition of all reagents and catalysts, acetonitrile (CH_3_CN) was used as the reaction solvent to achieve a total reaction volume of 2.0 mL. The reaction tubes were placed in an oil bath mounted on the RSLAB-4C digital hot plate stirrer. To ensure uniform heating of the reaction mixtures, the oil level was maintained above the solution level within the tubes. Reactions were carried out at 20, 40, and 80 °C for 2 h and at 20 °C for 15 min with continuous stirring at 600 rpm. After the desired time, the reaction mixtures were cooled to ambient temperature, and aliquots were prepared for the NMR analysis. Specifically, 200 μL of the crude reaction solution was diluted with 500 μL of CDCl_3_. For peak identification, pure reagents were analyzed. Reagents in amounts equivalent to those employed under the reaction conditions were diluted with 500 μL of CDCl_3_ prior to analysis. The resulting samples were analyzed by ^1^H NMR spectroscopy on a Bruker Avance III 400 MHz spectrometer. Product yields were determined from the ^1^H NMR spectra and expressed as the molar percentage of a product relative to the starting material. NMR spectra were recorded at 400 MHz for ^1^H and 100 MHz for ^13^C. Chemical shifts (δ) are reported in ppm and referenced to residual solvent signals (CDCl_3_: δH 7.26, δC 77.16). Signal multiplicities are designated as s (singlet) and m (multiplet). Spectra were processed using Bruker TopSpin 5.0.0. For species characterized in situ, reported chemical-shift ranges correspond to values observed in multiple reaction mixtures.

BDMA: ^1^H NMR (400 MHz, CDCl_3_): δ 7.51–7.34 (m, 5H), 5.43 (s, 1H), 3.35 (s, 6H); TMSCN: ^1^H NMR (400 MHz, CDCl_3_): δ 0.24 (s, 9H); BA: ^1^H NMR (400 MHz, CDCl_3_): δ 9.95 (s, 1H), 7.83–7.81 (m, 2H), 7.58–7.54 (m, 1H), 7.48–7.44 (m, 2H); PhCH­(OMe)­(CN): ^1^H NMR (400 MHz, CDCl_3_, in situ): δ 7.4–7.3 (m, 5H), 5.18–5.11 (s, 1H), 3.49–3.40 (s, 3H); PhCH­(CN)­(OTMS): ^1^H NMR (400 MHz, CDCl_3_, in situ): δ 7.37–7.28 (m, 5H), 5.45 (s, 1H), 0.10 (s, 9H).

## Results

### Active Catalytic Species

The coordination numbers four and six are the most typical for the Sn­(IV) center in solutions, although other coordination numbers (e.g., five) are also possible. Calculations indicated that the most stable structure of the hydrated tin­(IV) chloride in acetonitrile solution is the octahedral one *cis*-[SnCl_4_(H_2_O)_2_] (**1**, coordination number six) with the *cis*-configuration of the water ligands. The *trans*-isomer *trans*-[SnCl_4_(H_2_O)_2_] is by 3.6 kcal/mol less stable in terms of Δ*G*, while the penta-coordinated trigonal bipyramid complex *ax*-[SnCl_4_(H_2_O)]­(H_2_O) with the water ligand in an axial position is endoergic by 4.5 kcal/mol. The isomer *eq*-[SnCl_4_(H_2_O)]­(H_2_O) with water being in an equatorial position, as well as the tetrahedral complex [SnCl_4_]­(H_2_O)_2_, do not exist in solution; all attempts to find the equilibrium structures resulted in the formation of either *ax*-[SnCl_4_(H_2_O)]­(H_2_O) or [SnCl_4_(H_2_O)_2_].

Salts of Sn­(IV) in aqueous solutions undergo noticeable hydrolysis due to the pronounced acidic nature of water coordinated to the Sn­(IV) center. Our calculations indicate that the Gibbs free energy of reaction *cis*-[SnCl_4_(H_2_O)_2_]­(H_2_O) → *cis*-[SnCl_4_(OH)­(H_2_O)]­(H_3_O) in acetonitrile is 16.8 kcal/mol. The deeper hydrolysis accompanied by the elimination of one chloride ion is also not favorable, with the Δ*G* value of the reaction *cis*-[SnCl_4_(H_2_O)_2_]­(H_2_O)_2_ → *fac*-[SnCl_3_(OH)­(H_2_O)_2_]­(H_3_O)­(Cl) being 6.4 kcal/mol. Therefore, the most thermodynamically stable catalytic species in acetonitrile solution under neutral conditions is *cis*-[SnCl_4_(H_2_O)_2_]. This is coherent with previous experimental results, which demonstrated that introduction of water into the system SnCl_4_–acetone gives [SnCl_4_(H_2_O)_2_].^77,78^

### The C–OMe Bond Cleavage in Acetal and Formation of a Carbocation

The first step of the acetal transformation is a cleavage of the electrophilically activated C–OMe bond. The activation may be achieved by an electrophilic attack at the oxygen atom of the methoxy group that facilitates the C–OMe bond cleavage. In the conventional mechanism of acetal hydrolysis in an acidic medium,^79,80^ the acetal is activated by protonation of one of the alkoxy groups that provokes the alcohol elimination and nucleophilic attack at the carbon atom of the oxonium ion, affording hemiacetal (Scheme 4). Besides a proton, the acetal’s C–OR bond may also be activated by other electrophiles present in the reaction mixture such as the Sn­(IV) or Si­(IV) centers in our case.

**Scheme 4:**
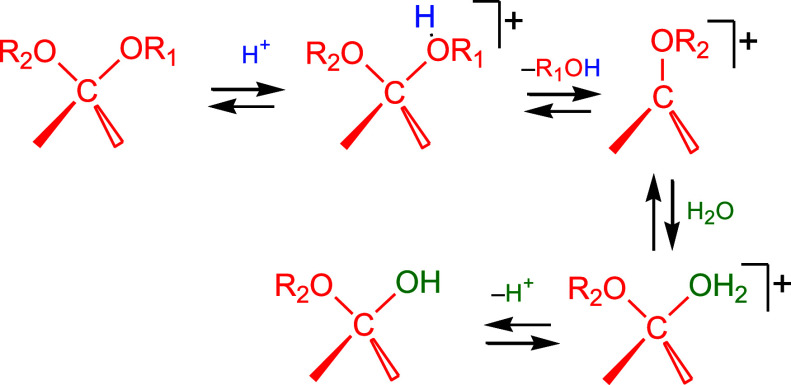
Conventional Mechanism of Acetal Hydrolysis in an Acidic Medium

#### Activation of the C–OMe Bond by Protonation (Pathway 1)

Due to the acidic character of water molecules coordinated to the Sn­(IV) center, a relatively facile *H*-transfer from the ligated H_2_O to one of the OMe groups of the acetal leads to the generation of the solvent-separated ion pair *cis*-[SnCl_4_(OH)­(H_2_O)]^−^···MeOH···[PhCH­(OMe)]^+^ (**2** ^**–**^···MeOH···[PhCH­(OMe)]^+^) via transition state **TS1** (Scheme 5). A quite low activation barrier (Δ*G* ^≠^ = 14.6 kcal/mol) indicates that this process is efficient under experimental conditions. Previously,^72^ some of us found that formation of the [PhCH­(OMe)]^+^···MeOH···[catalyst]^−^ ion pair assisted by a Ru­(II) cymene complex [Ru­(cym)­(L)­Cl] featuring a *N*,*N*-coordinating pyrazolone-based hydrazone ligand is noticeably facilitated by a water molecule, which is not directly involved in this step. In the case of the *cis*-[SnCl_4_(H_2_O)_2_] catalyst, the effect of water in this step is insignificant (see **TS1′** in Scheme S1, A in Supporting Information). Finally, the water-assisted intramolecular *H*-transfer in complex *cis*-[SnCl_4_(H_2_O)­{PhCH­(OMe)_2_}] via **TS1″** and the TMSCN-assisted *H*-transfer from the coordinated water molecule to the acetal via **TS1‴** (Scheme S1B,C) are also less favorable.

**Scheme 5:**
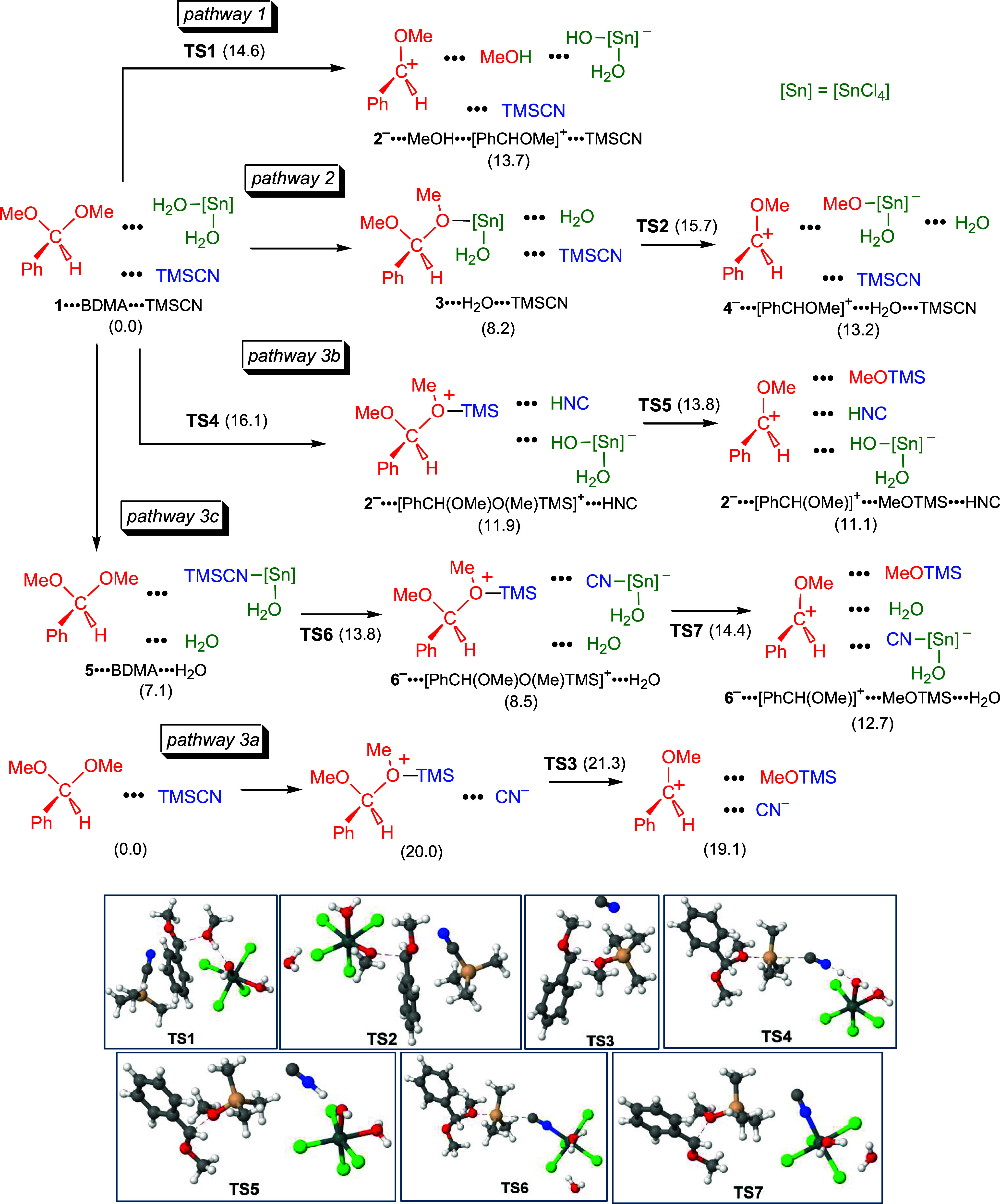
Mechanisms and Equilibrium Structures of the Corresponding Transition States for the Initial Activation of the Acetal and [PhCH­(OMe)]^+^ Formation (Relative Δ*G* Values in kcal/mol Are in Parentheses)

#### Activation of the C–OMe Bond by Coordination to Sn­(IV) (Pathway 2)

Another way of acetal activation is its coordination to the Sn­(IV) center of the catalysts. The substitution of one water ligand for the acetal molecule to give **3** (Scheme 5) is endoergic by 8.2 kcal/mol. The monomolecular C–OMe bond cleavage in complex **3** requires the activation barrier of 15.7 kcal/mol relative to the initial reactants, and formation of the ion pair **4** ^**–**^···[PhCH­(OMe)]^+^ is endoergic by 13.2 kcal/mol. In the presence of TMSCN, the C–OMe bond cleavage may be assisted by the nitrile molecule. This process is stepwise and includes formation of the *cis*-[SnCl_4_(H_2_O)­{O­(Me)­CH­(Ph)­O­(Me)­TMS}]^+^···CN^–^ ion pair (Scheme S2). However, the calculated activation barrier of the first step is too high for the realization of this route.

#### Activation of the C–OMe Bond by Silylation (Pathways 3)

The reaction between acetal and uncoordinated TMSCN (conditions corresponding to the uncatalyzed process) may afford the [PhCH­(OMe)­O­(Me)­TMS]^+^···CN^–^ ion pair (pathway 3a, Scheme 5). Analysis of the potential energy surface (PES) indicates that the energy of the acetal···TMSCN system increases monotonously upon the Si–C bond cleavage and the Si–O bond formation (Figure S1 in Supporting Information). The C–OMe­(TMS) bond rupture in [PhCH­(OMe)­O­(Me)­TMS]^+^ leads to [PhCH­(OMe)]^+^ and TMSOMe via **TS3** with the overall activation barrier of 21.3 kcal/mol.

In the presence of the catalyst, two silylation routes are possible. In the first one (pathway 3b, Scheme 5), the TMS group transfer is accompanied by the protonation of the CN group with the coordinated water molecule of the catalyst. This step is concerted, occurs via **TS4** with the activation barrier of 16.1 kcal/mol, and gives silylated acetal cation, complex *cis*-[SnCl_4_(OH)­(H_2_O)]^−^, and hydrogen isocyanide HNC. Subsequent C–O bond rupture affords the carbocation [PhCH­(OMe)]^+^ via **TS5**.

In the second route (pathway 3c, Scheme 5), TMSCN can coordinate to the Sn­(IV) center through the nitrile N atom. The substitution of one H_2_O molecule in *cis*-[SnCl_4_(H_2_O)_2_] for TMSCN to give *cis*-[SnCl_4_(H_2_O)­(NCTMS)] (**5**) requires 7.1 kcal/mol. The coordination activates the Si–CN bond toward its cleavage. Indeed, the calculated Si–C bond length in TMSCN increases upon its coordination from 1.901 to 1.929 Å, while the corresponding Wiberg bond index decreases from 0.75 to 0.71. The calculated vertical energy of the heterolytic Si–C bond cleavage in **5** is lower than that in free TMSCN by 16.0 kcal/mol. Such an activation was previously proposed upon TMSCN coordination to a Zr­(IV) center.^81^

After formation of **5**, the TMS group transfer occurs between **5** and the acetal in one step via **TS6** with the overall activation barrier of only 13.8 kcal/mol, yielding the silylated acetal cation and the isocyanide tin complex *cis*-[SnCl_4_(NC)­(H_2_O)]^−^ (**6** ^**–**^). The characteristic structural feature of **TS6** is the axial positions of the breaking and creating Si–C and Si–O bonds, respectively. It is important that the stabilization of the CN^–^ ion by its coordination to Sn­(IV) makes possible the easy TMS transfer, in contrast to the uncoordinated TMSCN (see above). The silylated acetal cation undergoes the C–O­(Me)­TMS bond rupture via **TS7** to form TMSOMe and the [PhCH­(OMe)]^+^ carbocation with the activation barrier of 14.4 kcal/mol. This is the rate-determining step of the carbocation formation through this pathway.

A similar pathway may be based on the tin hydroxo complex *cis*-[SnCl_4_(OH)­(H_2_O)]^−^ formed in pathway 1 (or, to a lesser extent, as a result of hydrolysis of *cis*-[SnCl_4_(H_2_O)_2_]). It includes the substitution of the water ligand for TMSCN (Δ*G* = 1.4 kcal/mol), reaction with acetal via **TS6′**, and the C–O­(Me)­TMS bond cleavage to give [PhCH­(OMe)]^+^ via **TS7′** (Scheme S3). However, this pathway is more energetically demanding (by 14.1 kcal/mol) than that involving **TS6** and **TS7**.

Finally, the activation pathway involving simultaneous coordination of both acetal and TMSCN to Sn­(IV) was found to be unfavorable (Scheme S4).

Analysis of the energetic characteristics indicates that pathways 1 and 3c are the most favorable ones for the C–OMe bond rupture in the acetal and the generation of carbocation [PhCH­(OMe)]^+^ (Δ*G^≠^* = 14.6 and 14.4 kcal/mol, respectively), and they should occur concurrently. Meanwhile, pathways 2 and 3b exhibit quite accessible activation barriers (Δ*G^≠^* = 15.7 and 16.1 kcal/mol, respectively) and, although to a lesser extent, should also contribute to the generation of [PhCH­(OMe)]^+^.

### Nucleophilic Attack at the [PhCH­(OMe)]^+^ Cation

The carbocation [PhCH­(OMe)]^+^ formed in the previous step bears a highly electrophilic sp^2^ carbon atom, which is a target for an attack by nucleophiles presented in the reaction mixture. First (pathway 4, Scheme 6), the carbocation and the hydroxo complex **2** ^**–**^ generated within pathway 1 can easily recombine via **TS8** to give directly a hemiacetal coordinated to the Sn­(IV) center (**7**). The hemiacetal undergoes a facile proton transfer assisted by a water molecule of the catalyst via **TS9** to give benzaldehyde as a stable product and the regenerated catalyst. Second (pathway 5), the nucleophilic addition of water to the carbocation [PhCH­(OMe)]^+^ toward hemiacetal via **TS10** is less favorable than the ion pair recombination.

**Scheme 6:**
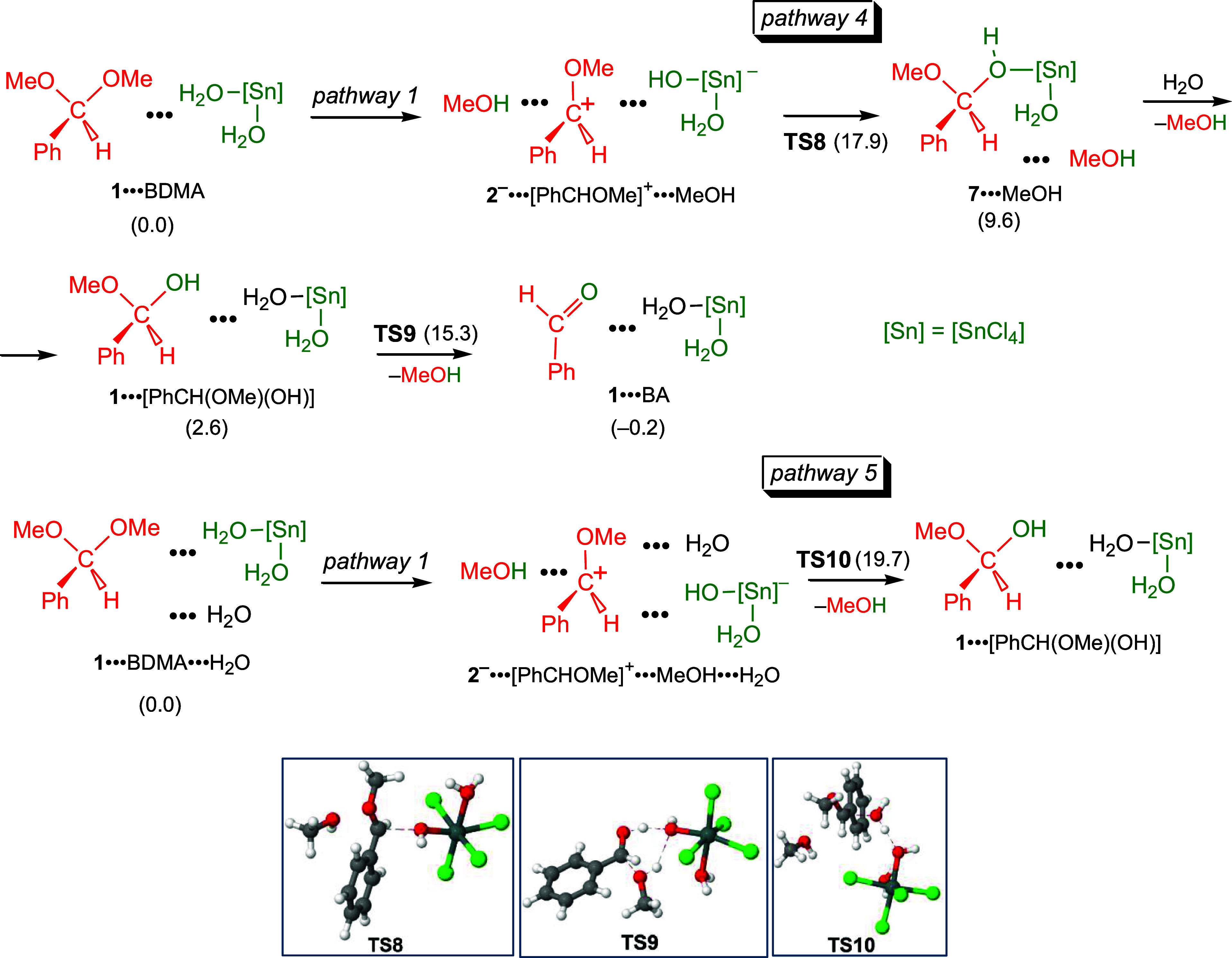
Pathways 4 and 5 of the Further Evolution of [PhCH­(OMe)]^+^ and Equilibrium Structures of the Corresponding Transition States (Relative Δ*G* Values in kcal/mol Are in Parentheses)

Third (pathway 6, Scheme 7), removal of TMSOMe to outside of the ion pair **6** ^**–**^···TMSOMe···[PhCH­(OMe)]^+^ of pathway 3c induces a recombination of the carbocation and **6** ^**–**^ via **TS11**, producing the final product PhCH­(OMe)­(CN) coordinated to the Sn­(IV) center.

**Scheme 7:**
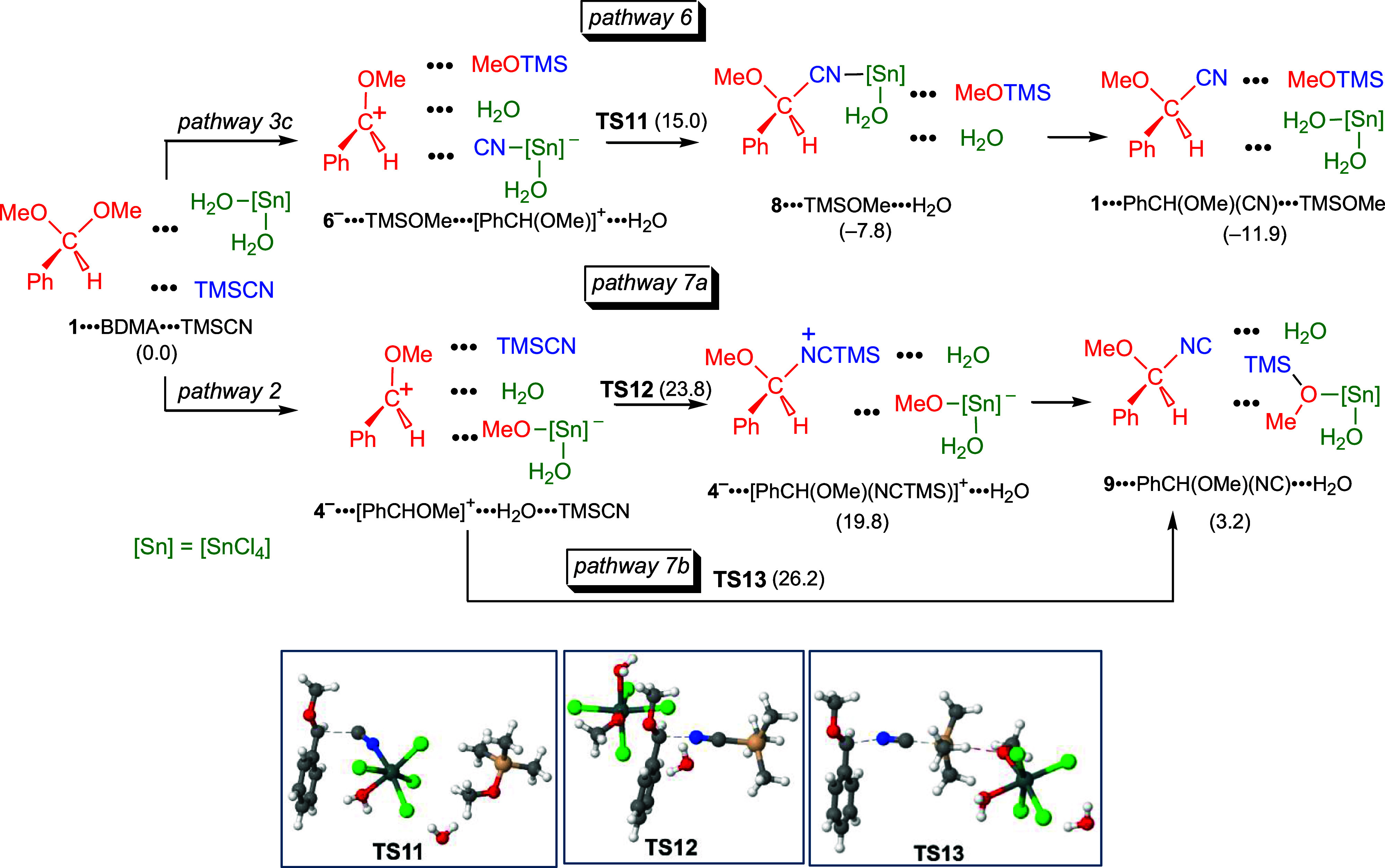
Pathways 6 and 7 of the Further Evolution of [PhCH­(OMe)]^+^ and Equilibrium Structures of the Corresponding Transition States (Relative Δ*G* Values in kcal/mol Are in Parentheses)

Fourth (pathways 7, Scheme 7), a free TMSCN molecule existing in the reaction mixture can also attack the carbocation by the N atom. This process can occur either in a stepwise or in a concerted manner. In the stepwise pathway 7a, the silylated isocyanide product [PhCH­(OMe)­(NCTMS)]^+^ is first formed via **TS12**. We were unable to find a TS for the following TMS transfer to the counterion **2** ^**–**^ or **4** ^**–**^. However, analysis of PES including the surface scan indicated that this process is spontaneous when the TMS group of the cation is oriented toward the anionic methoxy or hydroxy Sn complex (see Supporting Information for details). The concerted pathway 7b includes the simultaneous C–N and Si–O bond making and Si–C bond cleavage via **TS13**.

Fifth (pathway 8, Scheme 8), TMSCN can be initially decomposed upon reaction with **2** ^**–**^ (or **4** ^**–**^) to form a free CN^–^ anion via **TS14**. The cyanide anion reacts with [PhCH­(OMe)]^+^, producing the final reaction product PhCH­(OMe)­(CN). The latter step is almost barrierless, as indicated by the analysis of PES (see Supporting Information for details).

**Scheme 8:**
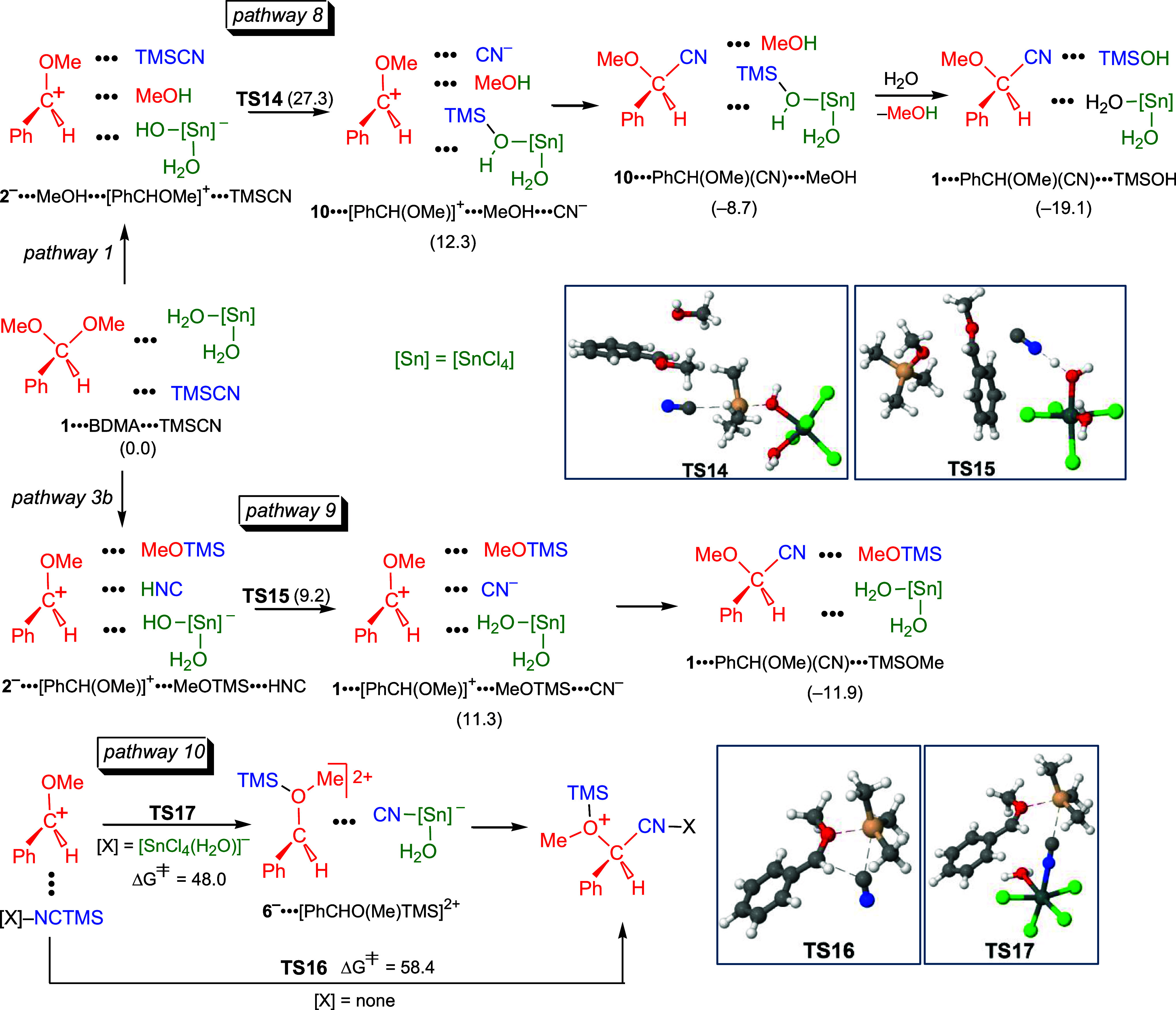
Pathways 8–10 of the Further Evolution of [PhCH­(OMe)]^+^ and Equilibrium Structures of the Corresponding Transition States (Relative Δ*G* Values in kcal/mol Are in Parentheses; the Gibbs Free Energies of Activation Are Indicated in kcal/mol for the Individual Steps of Pathway 10)

Sixth (pathway 9, Scheme 8), the hydrogen isocyanide HNC formed in pathway 3b may be easily deprotonated by the hydroxo complex **2** ^**–**^ via **TS15**, followed by the direct cyanation of [PhCH­(OMe)]^+^ by the CN^–^ anion with formation of PhCH­(OMe)­(CN) and regeneration of the catalyst.

Finally, seventh, the mechanism of cyanosilylation of carbonyl compounds described in the literature^42,56–61^ and including the Si–C bond cleavage in TMSCN and the TMS transfer to the O atom of the substrate (pathway 10) can also be applied to the carbocation [PhCH­(OMe)]^+^ bearing the central C atom in the sp^2^ hybridization (Scheme 8). This process involving free TMSCN and **TS16** has a very high activation barrier of 58.4 kcal/mol. The reaction of [PhCH­(OMe)]^+^ with the coordinated TMSCN occurs in a stepwise manner via **TS17** but still has a very high activation energy of 48.0 kcal/mol.

Thus, the main product of the reaction between benzaldehyde dimethyl acetal and TMSCN in the presence of Sn­(IV) chloride is 2-methoxy-2-phenylacetonitrile, PhCH­(OMe)­(CN), mostly formed through pathways 3c + 6 (total activation energy of 15.0 kcal/mol). Meanwhile, the generation of acetaldehyde as a side product is also expected via pathways 1 + 4 (total activation barrier of 17.9 kcal/mol). The isomeric α-methoxybenzyl isocyanide PhCH­(OMe)­(NC) may also be formed in a much lesser amount through pathways 1 (or 2) + 7 (total activation barrier of 23.8 kcal/mol).

### Isomerization of PhCH­(OMe)­(NC)

The α-methoxybenzyl isocyanide PhCH­(OMe)­(NC) mentioned above as an expected side product of the reaction under study can be easily isomerized in the presence of *cis*-[SnCl_4_(H_2_O)_2_] into the more thermodynamically stable PhCH­(OMe)­(CN) (pathway 11, Scheme 9). At the first step of the mechanism, substitution of one water ligand for PhCH­(OMe)­(NC) coordinated via the cyanide carbon atom occurs. Such a coordination activates the C–N bond toward its cleavage via **TS18** to give the ion pair *cis*-[SnCl_4_(H_2_O)­(CN)]^−^···[PhCH­(OMe)]^+^. Subsequent substitution of CN^–^ for H_2_O in the tin complex and the ion recombination lead to the final product PhCH­(OMe)­(CN). The C–N bond rupture step is rate-limiting and has a low activation barrier of 10.0 kcal/mol. Thus, any accumulation of PhCH­(OMe)­(NC) in the reaction mixture is not expected.

**Scheme 9:**
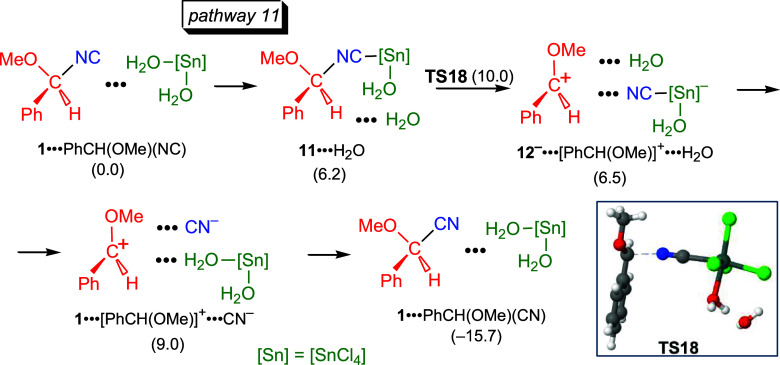
Mechanism of Isomerization of PhCH­(OMe)­(NC) into PhCH­(OMe)­(CN) and Equilibrium Structures of the Corresponding Transition States (Relative Δ*G* Values in kcal/mol Are in Parentheses)

### Evolution of Benzaldehyde

As was mentioned above, benzaldehyde is an expected side product in the reaction between acetal and TMSCN in the presence of *cis*-[SnCl_4_(H_2_O)_2_] due to a quite accessible overall activation barrier of 17.9 kcal/mol. Benzaldehyde can further undergo cyanosilylation with TMSCN in a well-known process, which typically occurs under catalytic conditions.^4,27–37^

In the presence of the catalyst [SnCl_4_(H_2_O)_2_], TMSCN is activated by its coordination to the Sn­(IV) center upon water ligand substitution to give *cis*-[SnCl_4_(H_2_O)­(NCTMS)] (**5**). The calculations indicated that the one-step cyanosilylation of PhCH­(=O) proposed in the literature^42,57–61^ is not possible with **5**, with no transition state of this type being located. Meanwhile, a stepwise mechanism for this process was found to be favorable, with the overall activation barrier of 13.4 kcal/mol (pathway 12, Scheme 10). It occurs via TMS transfer from **5** to PhCH­(=O) through **TS19**, both breaking and creating bonds being in the axial positions, to give the ion pair **6** ^**–**^···[PhCH­(OTMS)]^+^, which is then transformed into the cyanosilylated product **13** via **TS20**.

**Scheme 10:**
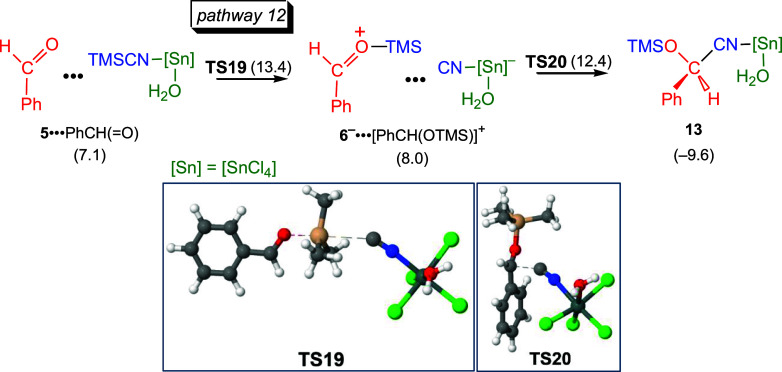
Mechanism of Cyanosilylation of Benzaldehyde and Equilibrium Structures of the Corresponding Transition States (Relative Δ*G* Values in kcal/mol Are in Parentheses)

Product **13** was stable under experimental conditions. Indeed, a possible way of its conversion into 2-methoxy-2-phenylacetonitrile includes the nucleophilic substitution of the OTMS group for the methoxy group, with the process being assisted by *H*-transfers with the involvement of [SnCl_4_(H_2_O)_2_] and a methanol molecule (Scheme 11). However, the calculated barrier of the first step (31.1 kcal/mol) is too high to permit efficient realization of this process.

**Scheme 11:**
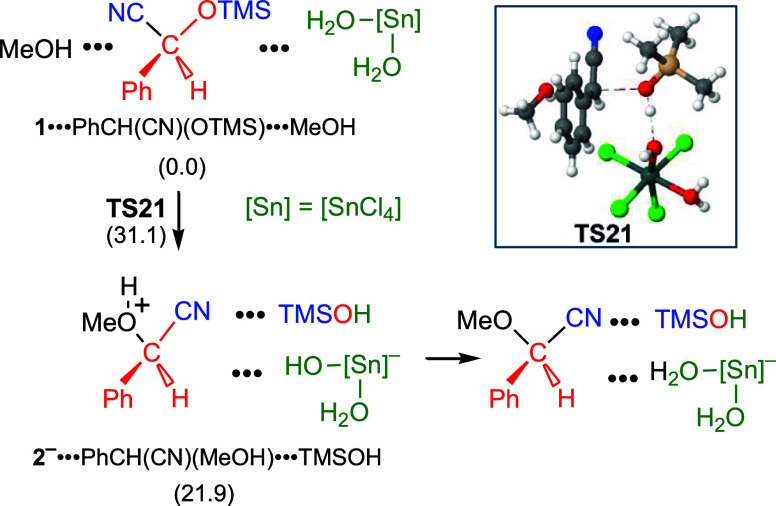
Mechanism of PhCH­(CN)­(OTMS) Transformation into PhCH­(OMe)­(CN) and the Equilibrium Structure of the Corresponding Transition State (Relative Δ*G* Values in kcal/mol Are in Parentheses)

### Formation and Evolution of HCN

The hydrogen cyanide HCN is obtained in situ upon the reaction of TMSCN with water or alcohols.^62,82–86^ Indeed, the calculated activation barrier of the bimolecular TMSCN hydrolysis with a water molecule occurring via **TS**_**HCN**_ (Scheme S5) is 25.5 kcal/mol that permits the realization of this process, at least to some extent, under experimental conditions.

Hydrogen cyanide is a known reagent used in cyanation reactions. Therefore, the involvement of HCN in the acetal cyanation was evaluated. Pathway 3c_HCN_—similar to pathway 3c found for TMSCN—includes substitution of a water ligand in the catalyst molecule for HCN, followed by the simultaneous proton transfer to the methoxy group of the acetal and cleavage of the C–OMe bond (Scheme 12). This route occurs via **TS22** and yields carbocation [PhCHOMe]^+^, complex **6** ^**–**^, and a methanol molecule.

**Scheme 12:**
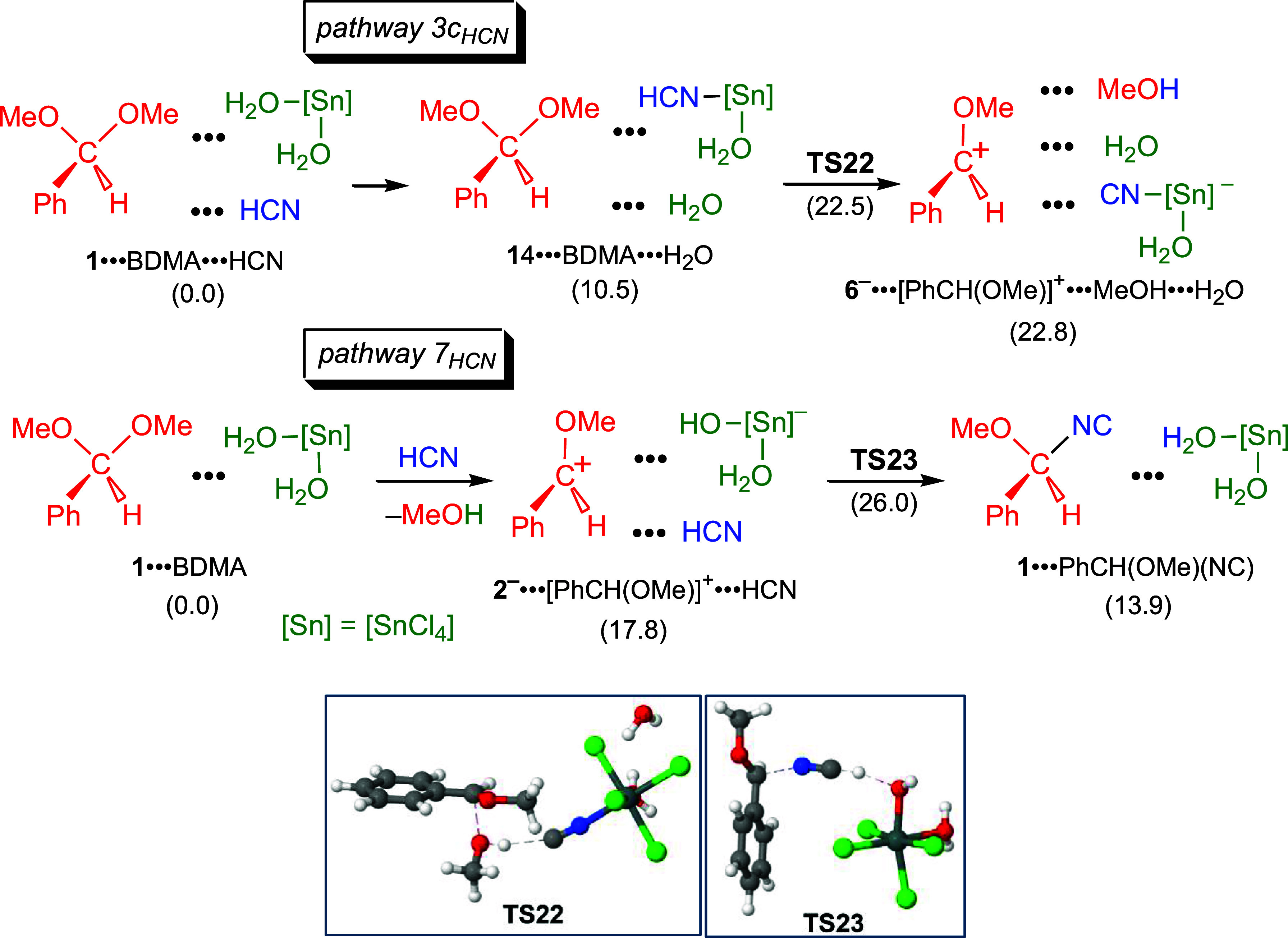
Mechanisms of Cyanation of BDMA with HCN and Equilibrium Structure of the Corresponding Transition State (Relative Δ*G* Values in kcal/mol Are in Parentheses)

Hydrogen cyanide can also serve as a nucleophile in the reaction with carbocation [PhCHOMe]^+^ (pathway 7_HCN_, Scheme 12). The nucleophilic addition occurs via **TS23** and includes a proton transfer to the hydroxo tin complex **2** ^**–**^ and formation of the α-methoxybenzyl isocyanide PhCH­(OMe)­(NC). The calculated activation energies of both pathways, 3c_HCN_ and 7_HCN_ (22.5 and 26.0 kcal/mol, respectively), are clearly higher than those of the corresponding pathways 3c and 7 with participation of TMSCN (14.4 and 23.8 kcal/mol, respectively). This is consistent with the higher H–CN bond energy compared to the Me_3_Si–CN bond energy. Thus, the primary cyanation agent in the system under study is TMSCN and not HCN formed in situ, and detection of HCN in the reaction mixture as a side product is quite expected.

As discussed above, the isomeric hydrogen isocyanide, HNC, generated in pathway 3b, should also readily react with [PhCH­(OMe)]^+^ (pathway 9), thus preventing its accumulation in the reaction mixture.

### Experimental Verification

To validate the theoretical predictions and confirm the proposed reaction mechanism, complementary experiments on the cyanation of acetals with TMSCN were conducted. Product identities and distributions were determined by ^1^H NMR spectroscopy based on the assignment and integration of diagnostic resonances of the reactants and products. First, the reaction between benzaldehyde dimethyl acetal (BDMA) and TMSCN in a molar ratio of 1:1 and in the presence of the catalyst SnCl_4_·5H_2_O in acetonitrile solution was analyzed at 80 °C, the temperature being the same as used in the previous work.^55^ After 2 h, ^1^H NMR analysis of the reaction mixture revealed the formation of PhCH­(OMe)­(CN) as a major product (85% yield; entry 1, Table 1, Figure S4). Characteristic signals at δ 3.43 and 5.12 ppm, corresponding to the methoxy (OCH_3_) and benzylic CH protons, respectively, confirmed the formation of PhCH­(OMe)­(CN). Additionally, benzaldehyde (BA) was observed as a minor product (15% yield), as indicated by the aldehydic proton resonance at δ 9.92 ppm. These results are completely coherent with theoretical predictions about formation of these two species, the cyanated product being the major one. Apparently, the available amount of TMSCN in this case is not sufficient for further cyanosilylation of BA through pathway 12, all cyanide being consumed in the cyanation of BDMA.

**Table 1:** Results of the ^1^H NMR Experiments

entry	reagents (mmol)	catalyst, mol %	products (yield %)^c^
1^a^	BDMA (1) + TMSCN (1)	1	PhCH(OMe)(CN) (85), BA (15)
2^b^	BDMA (1) + TMSCN (2)	1	PhCH(OMe)(CN) (100)
3^a^	BDMA (1)	1	BA (39)
4^a^	BA (1) + TMSCN (2)	1	PhCH(CN)(OTMS) (100)
5^a^	TMSCN (1)	1	TMSOH (63)
6^a^	BDMA (1) + TMSCN (2)	0	PhCH(OMe)(CN) (18), BA (19)

a Conditions: 80 °C, 2 h.

b Conditions: 20 °C, 40 °C, 80 °C; 15 min, 30 min, 45 min, 1 h, 1.5 h, 2 h (for each temperature). In all cases, BDMA is completely converted into PhCH­(OMe)­(CN).

c Determined by ^1^H NMR analysis (see the Experimental Details section).

When TMSCN was added in excess (the BDMA/TMSCN molar ratio of 1:2), the acetal was completely converted into PhCH­(OMe)­(CN), with no ^1^H NMR signals from BDMA and BA being detected (entry 2, Table 1, Figure S5, see also ref 55). Since TMSCN is directly involved in pathways 3c and 6, leading to PhCH­(OMe)­(CN), and it is not involved in pathways 1 and 4 toward BA, its excess shifts the equilibrium to the formation of the cyanated product, thus suppressing the formation of benzaldehyde. Hence, the theoretically predicted competition between two reaction channels (pathways 3c + 6 and pathways 1 + 4) is confirmed by the experimental data.

The acetal BDMA in the presence of the catalyst SnCl_4_·5H_2_O but without addition of TMSCN is partially converted into BA (yield 39%), the 1H resonances of BDMA and BA being presented at δ 5.32 and 9.98 ppm, respectively, with the area ratio of 1:0.65 (entry 3, Table 1, Figure S6). This observation additionally proves that the formation of BA occurs in a channel separate from cyanation (pathways 1 + 4) without the involvement of TMSCN.

To analyze the further evolution of benzaldehyde, the reaction between BA and TMSCN (1:2 molar ratio) in the presence of SnCl_4_·5H_2_O was also evaluated under the same conditions. As a result of this reaction, the ^1^H NMR signal of BA completely disappears and new signals at δ 0.10, 5.45, and 7.37–7.28 ppm assigned to Si­(CH_3_)_3_, benzylic CH, and aromatic protons, respectively,^87^ become visible (entry 4, Table 1, Figure S7). Thus, BA is completely converted into the cyanosilylated product [PhCH­(CN)­(OTMS). These results confirm the theoretically predicted cyanosilylation of BA via pathway 12 with a low activation barrier of 13.4 kcal/mol.

The TMSCN reagent is efficiently converted into TMSOH and HCN in the presence of the catalyst SnCl_4_·5H_2_O, as evidenced by the characteristic ^1^H NMR resonances at δ −0.02 ppm (Si­(CH_3_)_3_) and δ 4.03 ppm, respectively (entry 5, Table 1, Figure S8). This confirms the theoretically proposed activation of TMSCN by its coordination to the Sn­(IV) center toward the Si–C bond rupture predicted as the key step within pathway 3c.

In the blank experiment (BDMA + TMSCN in the ratio 1:2 without the catalyst), the formation of both PhCH­(OMe)­(CN) and BA was detected in almost equal proportions (18–19%, characteristic resonances at δ 3.49 (OCH_3_) and 5.18 (1H) ppm for PhCH­(OMe)­(CN) and δ 9.98 ppm (1H) for BA, entry 6, Table 1, Figure S9). Both these products are formed via the carbocation [PhCHOMe]^+^ generated through the uncatalyzed pathway 3a (Scheme 5) and following nucleophilic attack either by water or by CN^–^. The higher activation barrier of the uncatalyzed pathway 3a compared to the catalyzed pathways 1 and 3c (21.3 kcal/mol vs 13.7 and 14.4 kcal/mol) explains the lower product yields in the blank experiment compared to the catalytic ones.

The reaction between BDMA and TMSCN (1:2 molar ratio) in the presence of SnCl_4_·5H_2_O was also evaluated at lower temperatures, i.e., 20 °C and 40 °C, and for the shorter reaction time periods (15 min, 30 min, 45 min, 1 h, and 1.5 h). In all these experiments, BDMA was fully converted into the final cyanated product PhCH­(OMe)­(CN), the results being the same as those observed for 80 °C and 2 h of reaction time (entry 2, Table 1, Figures S10). This is in qualitative agreement with the low calculated value for the overall Gibbs free energy of activation of the cyanation reaction (15.0 kcal/mol), which should permit the fast realization of this process already at room temperature.

Finally, in all NMR spectra of products of the reactions in the presence of TMSCN, a signal coming from HCN at δ 4.03–4.16 ppm is detected. This confirms the easy formation of this species and its accumulation due to lower activity as the cyanation agent compared to TMSCN, as predicted by the theoretical calculations.

## Discussion

The DFT calculations confirmed by experimental data revealed two principal reaction channels in this system. The first major one is a cyanation of the acetal with TMSCN toward the major final reaction product PhCH­(OMe)­(CN), catalyzed by the *cis*-[SnCl_4_(H_2_O)_2_] active catalytic species. The most favorable mechanism of this process includes substitution of a water ligand in the catalyst molecule for TMSCN, the TMS transfer to an acetal molecules, the C–O_TMS_ bond rupture to give the key carbocation intermediate [PhCH­(OMe)]^+^ and the *cis*-[SnCl_4_(NC)­(H_2_O)]^−^ complex, and the ion recombination finally affording PhCH­(OMe)­(CN) and the initial catalyst (pathways 3c + 6, Schemes 5 and 7). In the concurrent less favorable mechanism, the TMS transfer between free TMSCN and acetal is accompanied by simultaneous protonation of the nitrile group by a coordinated water molecule to give HNC, followed by the C–O_TMS_ bond cleavage and the direct cyanation of [PhCH­(OMe)]^+^ by HNC (pathways 3b + 9, Schemes 5 and 8).

The second minor reaction channel is the tin-catalyzed hydrolysis of the acetal into benzaldehyde, which represents a minor reaction product. The mechanism of this process includes the initial protonation of the acetal with the coordinated water molecule, formation of the [PhCH­(OMe)]^+^···*cis*-[SnCl_4_(OH)­(H_2_O)]^−^ ion pair and methanol, the ion recombination to give the hemiacetal, and catalytic *H*-transfer leading to benzaldehyde (pathways 1 + 4, Schemes 5 and 6).

The further evolution of benzaldehyde as well as other intermediates formed via secondary pathways (i.e., PhCH­(OMe)­(NC) and HCN) was evaluated. When TMSCN is in excess, benzaldehyde is readily converted into the cyanosilylated product PhCH­(CN)­(OTMS) through concerted TMS transfer from the tin­(IV) coordinated TMSCN to the aldehyde, followed by cyanation of the [PhCH­(OTMS)]^+^ intermediate (pathway 12, Scheme 10). The α-methoxybenzyl isocyanide PhCH­(OMe)­(NC) totally isomerizes into the final nitrile product PhCH­(OMe)­(CN) via pathway 11 (Scheme 9), whereas the hydrogen cyanide HCN was revealed as a weaker cyanation agent compared to TMSCN, and therefore, it is accumulated in the reaction mixture.

## Conclusions

In this work, a detailed investigation of the reaction mechanism in the system benzaldehyde dimethyl acetal (PhCH­(OMe)_2_)/trimethylsilylcyanide (TMSCN)/SnCl_4_·5H_2_O of high practical potential has been carried out by both theoretical (DFT) and experimental (^1^H NMR) methods. The reaction proceeds predominantly through a cyanation pathway leading to the formation of α-methoxybenzyl cyanide, while acetal hydrolysis to benzaldehyde constitutes a minor competing process. The computational results are consistent with the experimental observations and identify the key intermediates and catalytic species involved in both reaction channels.

The study also demonstrates the crucial role of the tin­(IV) catalyst in both principal reaction channels. In the cyanation of acetal, the TMSCN coordination to the Sn­(IV) center activates this species toward the Si–C bond cleavage due to a shift of electron density from the nitrile group to the metal and stabilization of the CN^–^ anion in the complex *cis*-[SnCl_4_(NC)­(H_2_O)]^−^. In the acetal hydrolysis, the highly acidic nature of water coordinated to the Sn­(IV) center facilitates the acetal protonation and the C–O bond cleavage as well as the proton transfer in the hemiacetal—the key steps in the formation of benzaldehyde.

Overall, these findings provide detailed insight into the reactivity of acetals toward TMSCN in the presence of a strong Lewis acid such as the Sn­(IV) center and contribute to a broader understanding of catalytic cyanation processes.

## Supplementary Material





## Data Availability

The data underlying this study are available in the published article and its Supporting Information.

## Notes

Atomic coordinates, total and Gibbs free energies, and number of imaginary frequencies for the calculated equilibrium structures (XYZ)
Less favorable pathways, discussion of specific reaction steps, results of the relaxed PES scans, and ^1^H NMR spectra (PDF)
