# Supplementary material for: Strecker-Type Cyanation of Acetals Catalyzed by Tin(IV) Chloride—A Mechanistic Study
Source: J Org Chem. 2026 Jun 23;91(26):9045–57. doi: 10.1021/acs.joc.6c00737 (PMC13339649; doi:10.1021/acs.joc.6c00737)
Supplement: Supplementary file 2 [file jo6c00737_si_002.pdf]

Supporting Information

Strecker-type cyanation of acetals catalyzed by tin(IV) chloride – a mechanistic study

Ismayil M. Garazade,[†,‡] Armando J. L. Pombeiro,[†] and Maxim L. Kuznetsov*,[†]

[†] Centro de Química Estrutural, Institute of Molecular Sciences, Instituto Superior Técnico, Universidade de Lisboa, Av. Rovisco Pais, 1049-001 Lisbon, Portugal

[‡] Baku State University, Z. Xalilov Str. 23, AZ 1148 Baku, Azerbaijan

E-mail: max@mail.ist.utl.pt (M.L.K.).

Index

**Scheme S1.** Less favorable pathways of the initial acetal activation and equilibrium structures of the corresponding transition states	S2
**Scheme S2**. The C–O_{Me} bond cleavage assisted by TMSCN	S2
**Scheme S3.** Mechanism of silylation of the acetal catalyzed by the tin hydroxo complex *cis*-$[SnCl_4(OH)(H_2O)]^-$	S3
**Scheme S4.** Effect of the mutual coordination of the reactants on the activation of acetal	S3
**Scheme S5.** Mechanism of hydrolysis of TMSCN	S3
**Figure S1.** Relaxed PES scan of the structure $PhCH(OMe)_2\cdots$TMSCN toward the Si–C bond cleavage.	S4
**TMS transfer in 4$^-$•••$[PhCH(OMe)(NCTMS)]^+$**	S4
**Figure S2.** Optimization of the **4$^-$**•••$[PhCH(OMe)(NCTMS)]^+$ structure with TMS initially faced to the OMe group (A) and relaxed PES scan of **9**•••PhCH(OMe)(NC) toward the Si–C bond formation (B).	S5
**Ion recombination in 10•••$[PhCH(OMe)]^+$•••CN^-**	S5
**Figure S3.** Relaxed PES scan of **10**•••$[PhCH(OMe)]^+$•••CN^- toward the increase of the Si–N–C angle.	S6
**Figures S4–S14.** ^{1}H NMR spectra	S6

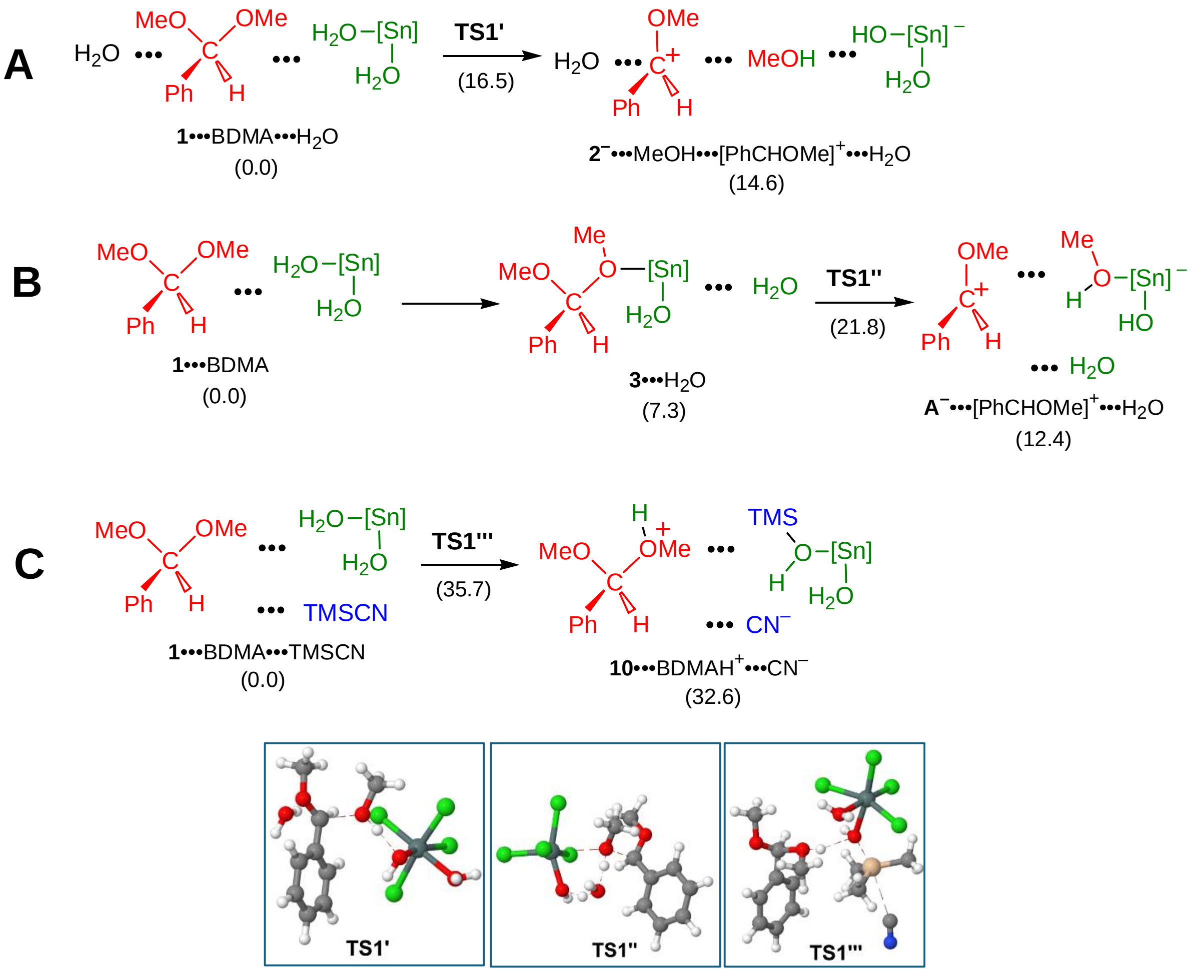

Scheme S1. Less favorable pathways of the initial acetal activation and equilibrium structures of the corresponding transition states (relative ΔG values in kcal/mol are in parentheses).

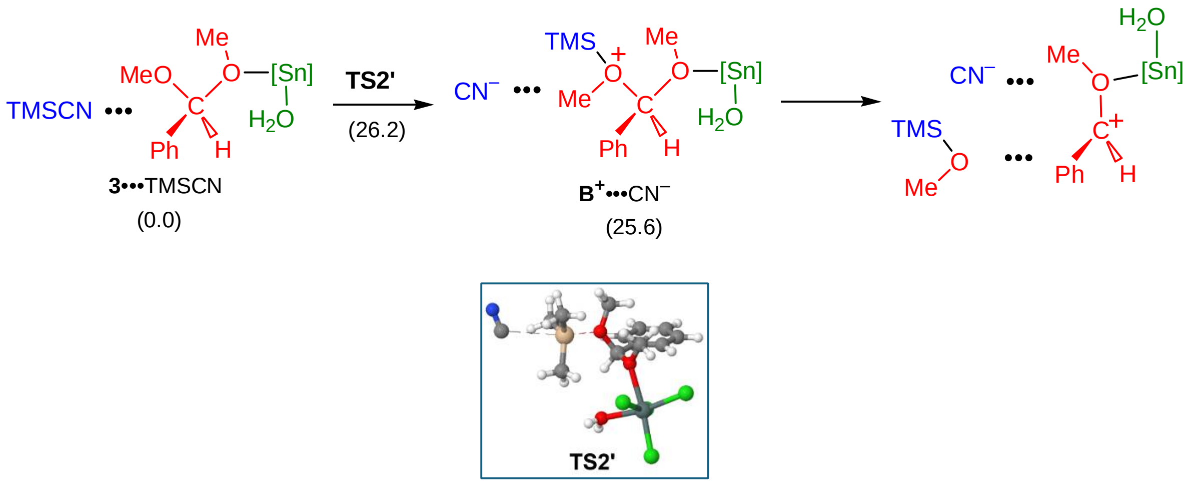

Scheme S2. The C–O_{Me} bond cleavage assisted by TMSCN (relative ΔG values in kcal/mol are in parentheses).

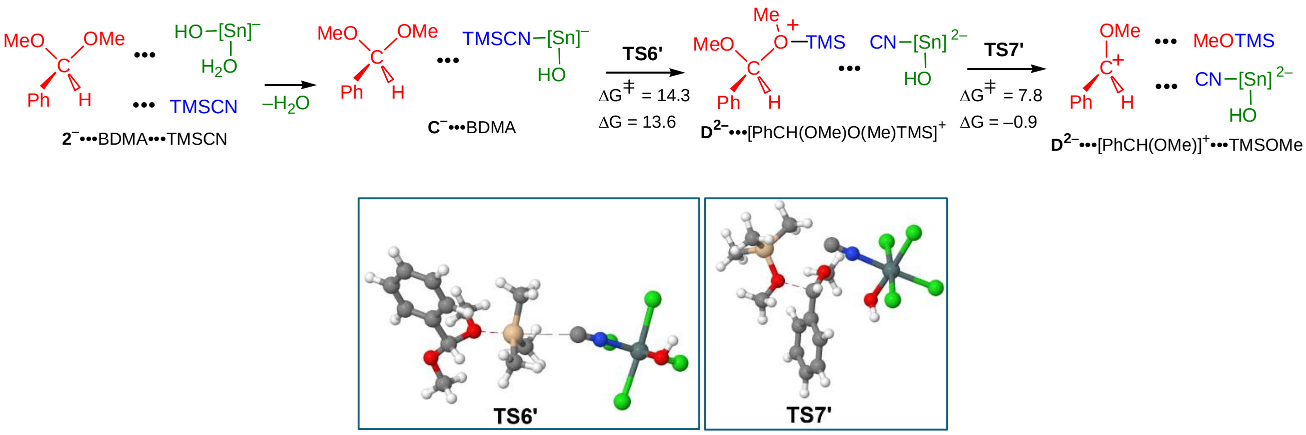

Scheme S3. Mechanism of silylation of the acetal catalyzed by the tin hydroxo complex *cis*-$[SnCl_4(OH)(H_2O)]^-$ (the Gibbs free energies of activation and reaction are indicated in kcal/mol for the individual steps).

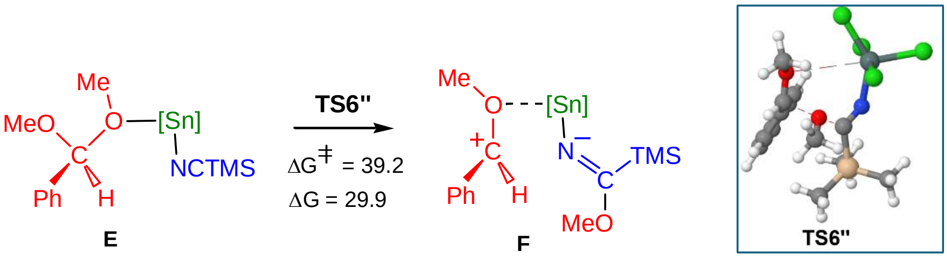

Scheme S4. Effect of the mutual coordination of the reactants on the activation of acetal (the Gibbs free energies of activation and reaction are indicated in kcal/mol).

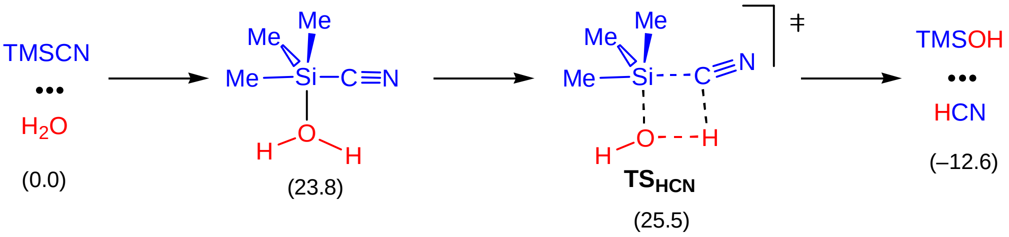

Scheme S5. Mechanism of hydrolysis of TMSCN (relative ΔG values in kcal/mol are in parentheses).

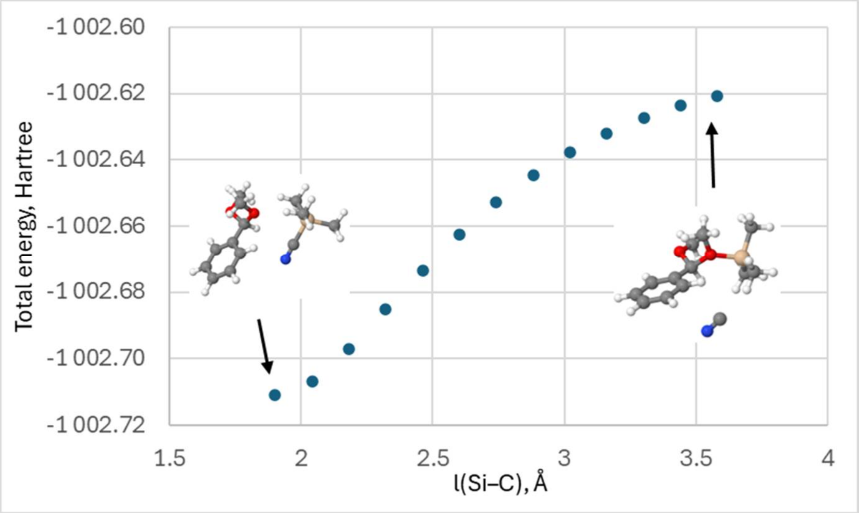

Figure S1. Relaxed PES scan of the structure $PhCH(OMe)_2$···TMSCN toward the Si–C bond cleavage.

TMS transfer in 4⁻•••[PhCH(OMe)(NCTMS)]⁺

We were unable to locate a transition state for the TMS transfer in the **4**⁻··· $[PhCH(OMe)(NCTMS)]^+$ species. To analyze the possibility of this process, we constructed an initial structure bearing two ions **4**⁻ and $[PhCH(OMe)(NCTMS)]^+$ so that the TMS group was faced to the OMe group of the tin complex with the initial Si···O distance of 4 Å. As a result of the geometry optimization, a spontaneous TMS transfer from $[PhCH(OMe)(NCTMS)]^+$ to **4**⁻ occurs (Figure S2, A). Additionally, the relaxed PES scan in **9**···PhCH(OMe)(NC) toward the Si–C bond formation revealed the monotonous energy decrease for the TMS transfer from $[PhCH(OMe)(NCTMS)]^+$ to **4**⁻ (Figure S2, B).

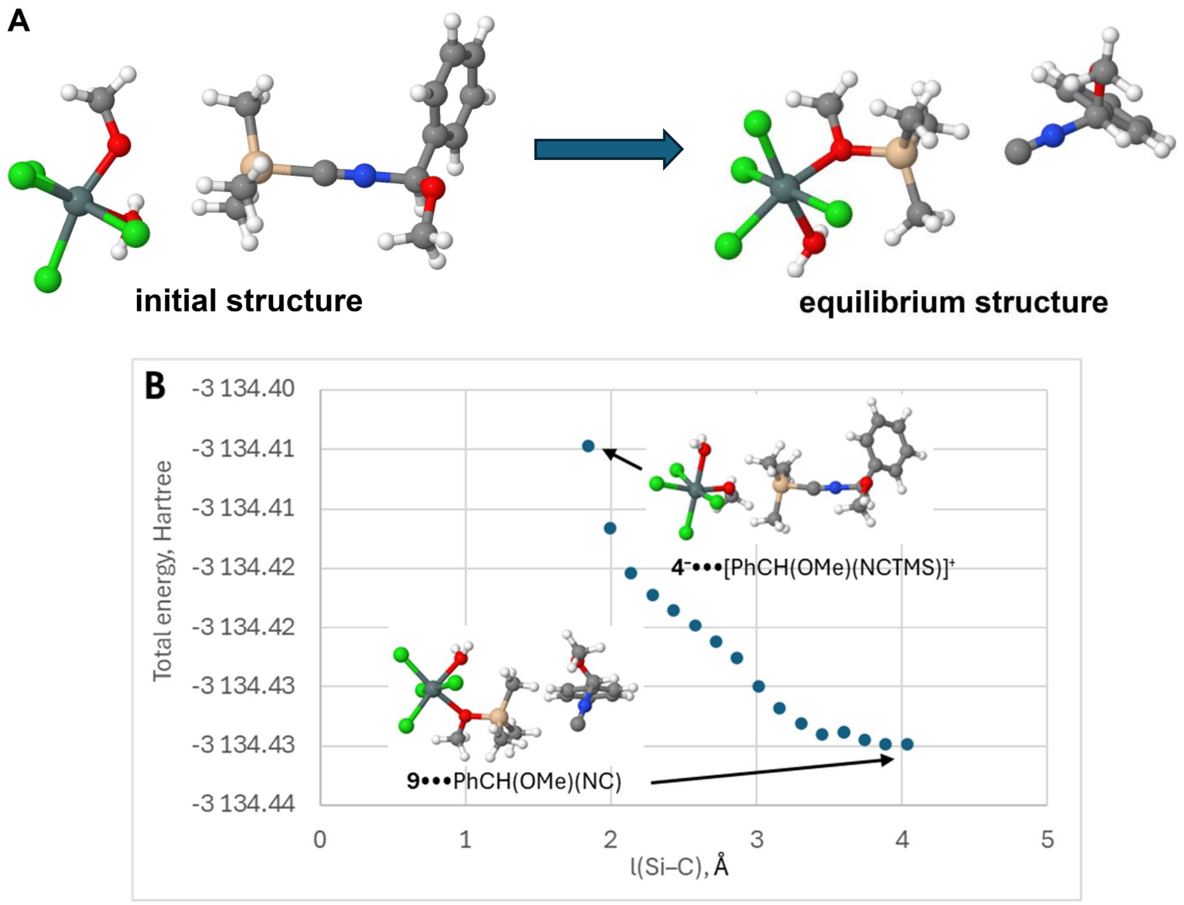

Figure S2. Optimization of the **4**$^{-}$•••[PhCH(OMe)(NCTMS)]$^{+}$ structure with TMS initially faced to the OMe group (A) and relaxed PES scan of **9**•••PhCH(OMe)(NC) toward the Si–C bond formation (B).

Ion recombination in 10···[PhCH(OMe)]$^{+}$···CN^{-}

To analyze the recombination of the [PhCH(OMe)]$^{+}$ and CN^{-} ions in **10**···[PhCH(OMe)]$^{+}$···CN^{-}, the relaxed PES scan of the later structure toward the increase of the SiNC angle (the CN^{-} rotation) was carried out. The calculations (Figure S3) indicate that the rotation of the CN^{-} anion, so that the carbon end becomes directed to the carbocation, results in almost spontaneous formation of PhCH(OMe)(CN).

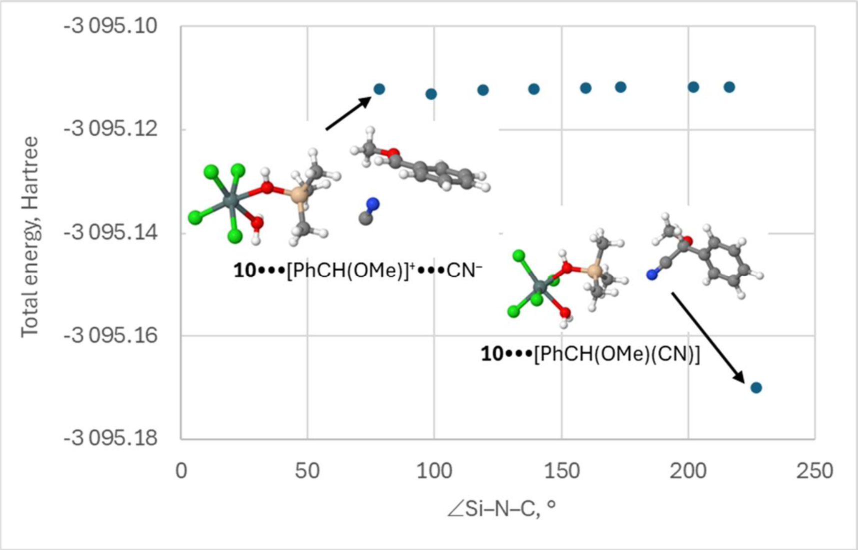

Figure S3. Relaxed PES scan of **10**···$[PhCH(OMe)]^{+}$···CN^{-} toward the increase of the Si–N–C angle.

The *cis*-$[SnCl_4(H_2O)(NC)]^{-}$ → *cis*-$[SnCl_4(H_2O)(CN)]^{-}$ isomerization

The calculations indicated that complex *cis*-$[SnCl_4(H_2O)(CN)]^{-}$ is by 3.4 kcal/mol more thermodynamically stable than *cis*-$[SnCl_4(H_2O)(CN)]^{-}$ (**6**$^{-}$) due to softer nature of the C donor center. However, the activation energy of the **6**$^{-}$ → **I** isomerization (15.1 kcal/mol via **TSI**) is significantly higher than the activation energy of the evolution of **6**$^{-}$ into the final product PhCH(OMe)(CN) along the pathways 3c + 6 (6.5 kcal/mol). Thus, intermediate **6**$^{-}$ once formed upon the TMSCN coordination does not isomerize into the CN-intermediate but is transformed into the final reaction product.

Application of $Al(H_2O)_6]^{3+}$ as a catalyst

Thermodynamic calculations of the most favorable pathways 3c and 6 for were also performed for $[Al(H_2O)_6]^{3+}$ used as a catalyst. The results indicate that the same mechanism found to be the most favorable for $SnCl_4$•$5H_2O$ is even more thermodynamically feasible for $[Al(H_2O)_6]^{3+}$, the least stable intermediate $\mathbf{5}_{\mathbf{Al}}$•••BDMA•••H_2O being less stable than the initial reagents $\mathbf{1}_{\mathbf{Al}}$•••BDMA•••TMSCN only by 4.4 kcal/mol (Scheme S6).

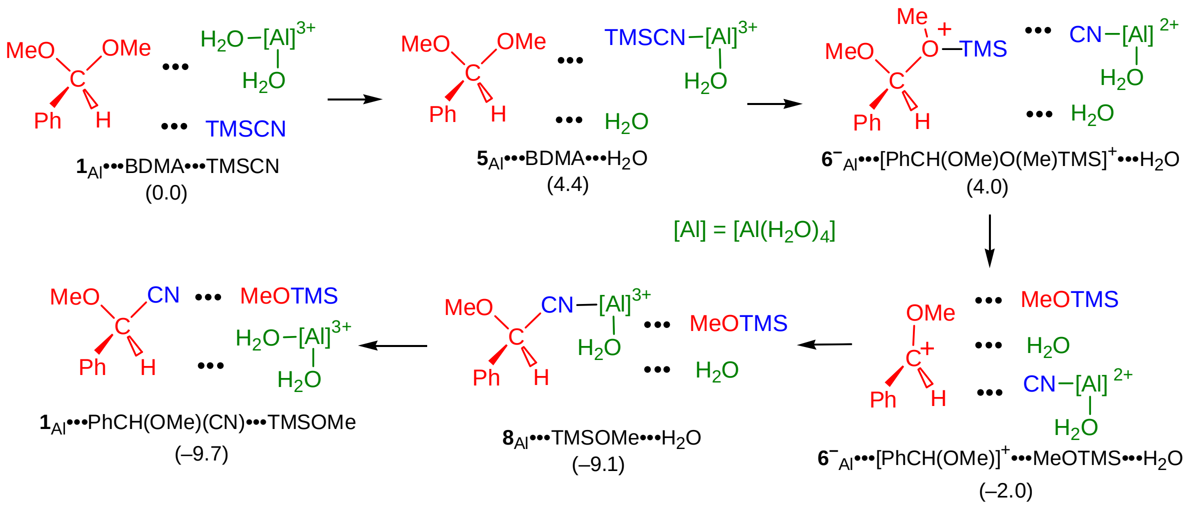

Scheme S6. Pathways 3c and 6 calculated for $[Al(H_2O)_6]^{3+}$ used as a catalyst.

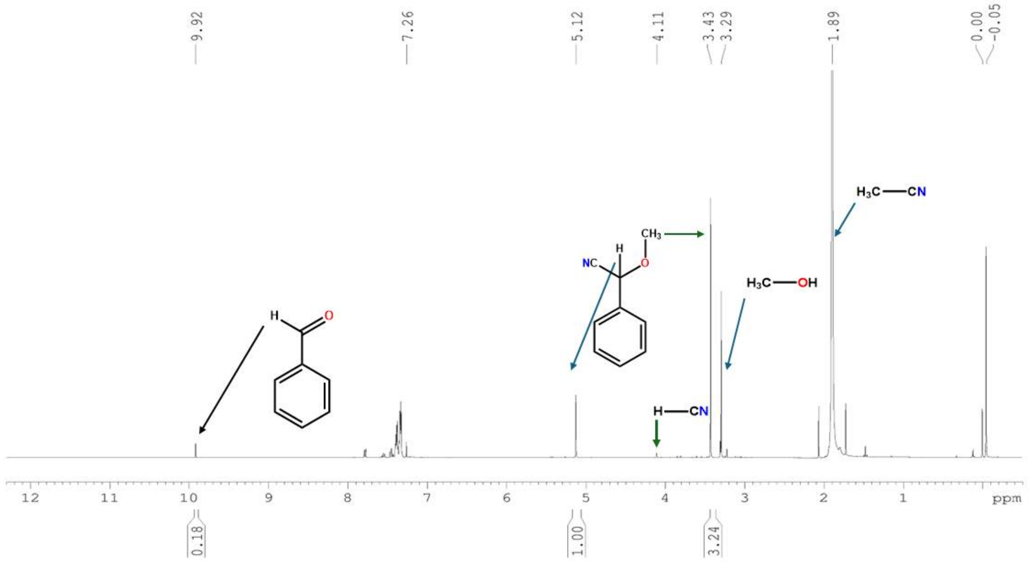

Figure S4. ^{1}H NMR (400 MHz, $CDCl_3$) spectrum of products of the reaction between BDMA and TMSCN (1 : 1) catalyzed by $SnCl_4$•$5H_2O$ (BA Yield (%) = (0.18) / (0.18 + 1.00) × 100 = 15 %).

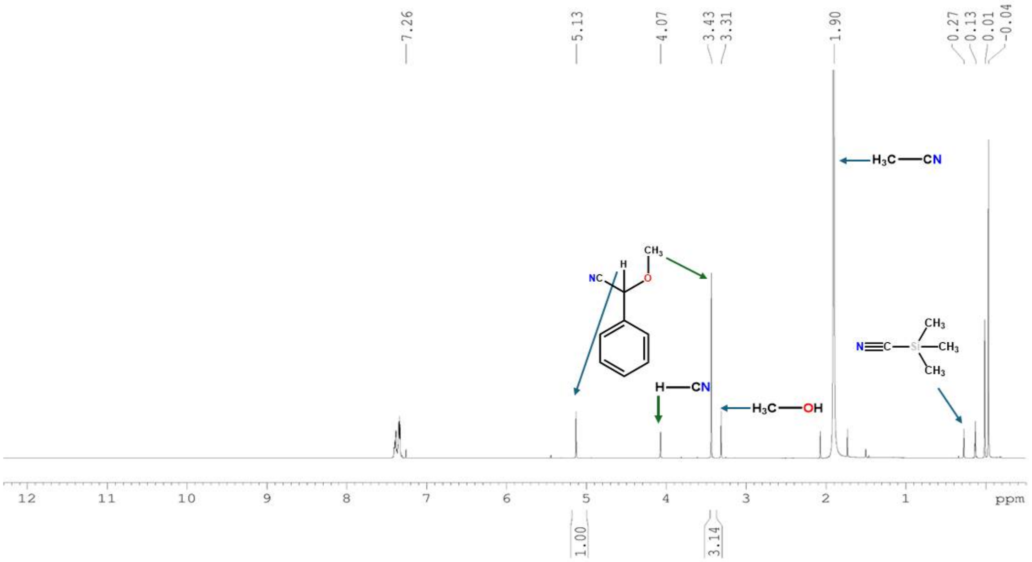

Figure S5. ^{1}H NMR (400 MHz, $CDCl_3$) spectrum of products of the reaction between BDMA and TMSCN (1 : 2) catalyzed by $SnCl_4$•$5H_2O$ (PhCH(OMe)(CN) Yield (%) = 100 %).

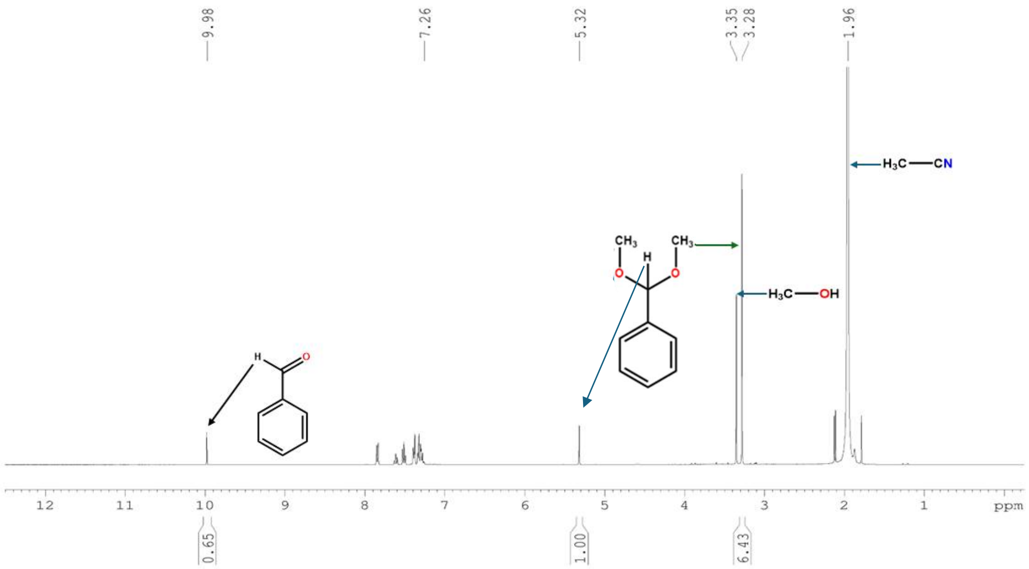

Figure S6. ^{1}H NMR (400 MHz, $CDCl_3$) spectrum of products of hydrolysis of BDMA in the presence of $SnCl_4$•$5H_2O$ (BA Yield (%) = (0.65) / (0.65 + 1.00) × 100 = 39 %).

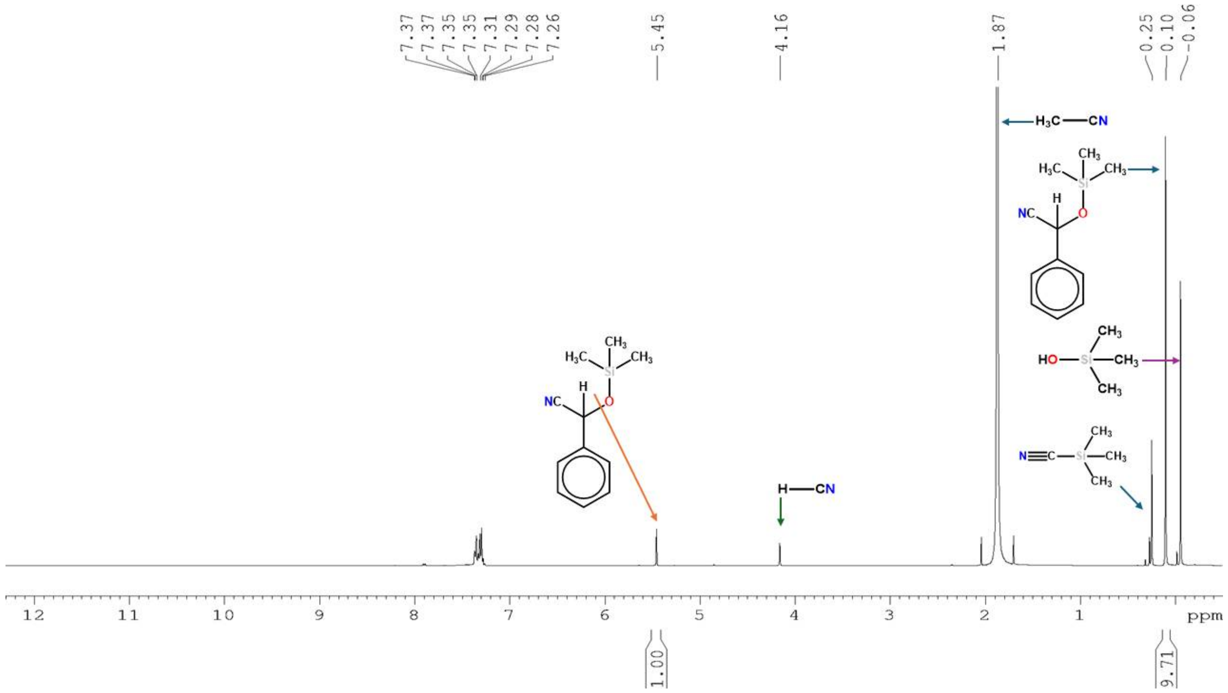

Figure S7. ^{1}H NMR (400 MHz, $CDCl_3$) spectrum of products of reaction of BA with TMSCN (1 : 2) catalyzed by $SnCl_4$•$5H_2O$ (PhCH(CN)(OTMS) Yield (%) = 100 %).

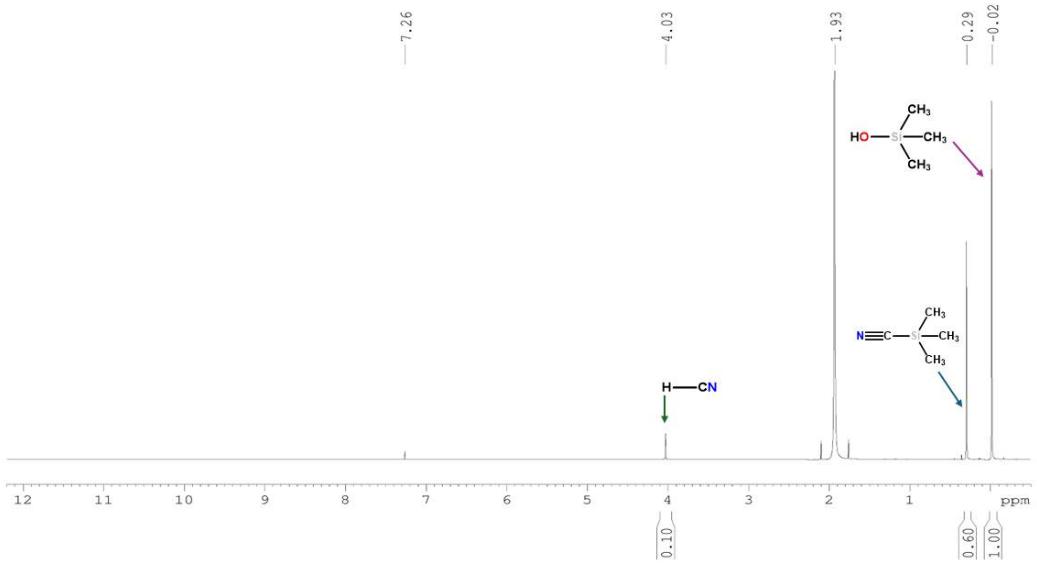

Figure S8. ^{1}H-NMR (400 MHz, $CDCl_3$) spectrum of products of hydrolysis of TMSCN in the presence of $SnCl_4$•$5H_2O$.

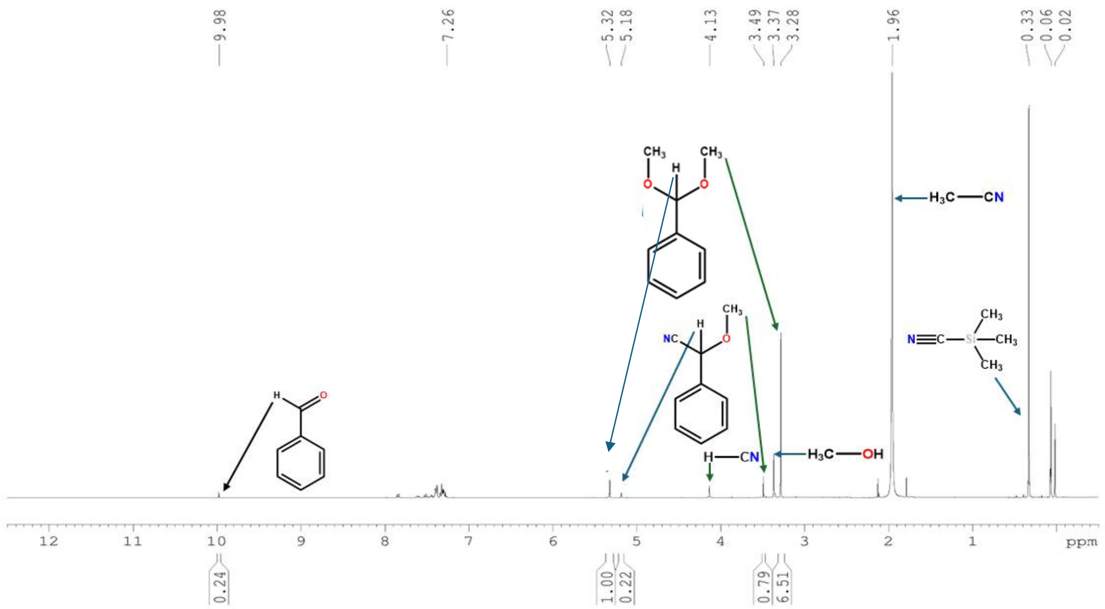

Figure S9. ^{1}H NMR (400 MHz, $CDCl_3$) spectrum of products of uncatalyzed reaction between BDMA and TMSCN (PhCH(OMe)(CN) Yield (%) = (0.22) / (0.22 + 1.00) × 100 = 18 %, BA Yield (%) = (0.24) / (0.24 + 1.00) × 100 = 19 %).

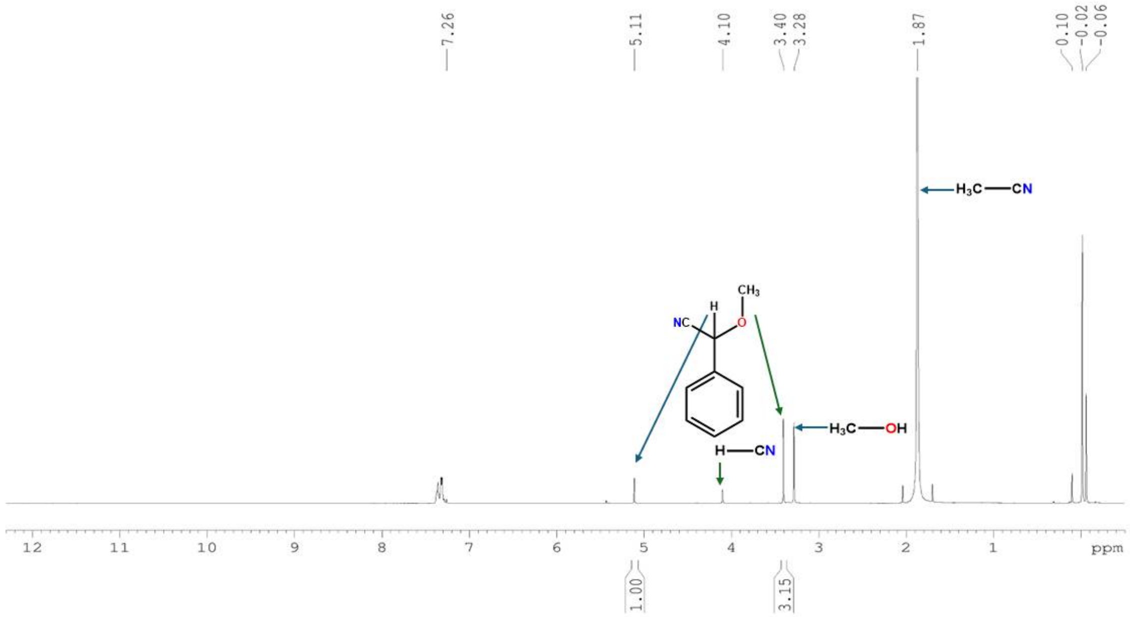

Figure S10. ^{1}H NMR (400 MHz, $CDCl_3$) spectrum of products of the reaction between BDMA and TMSCN (1 : 2) catalyzed by $SnCl_4$•$5H_2O$ at 20 °C after 15 min (PhCH(OMe)(CN) Yield (%) = 100 %).

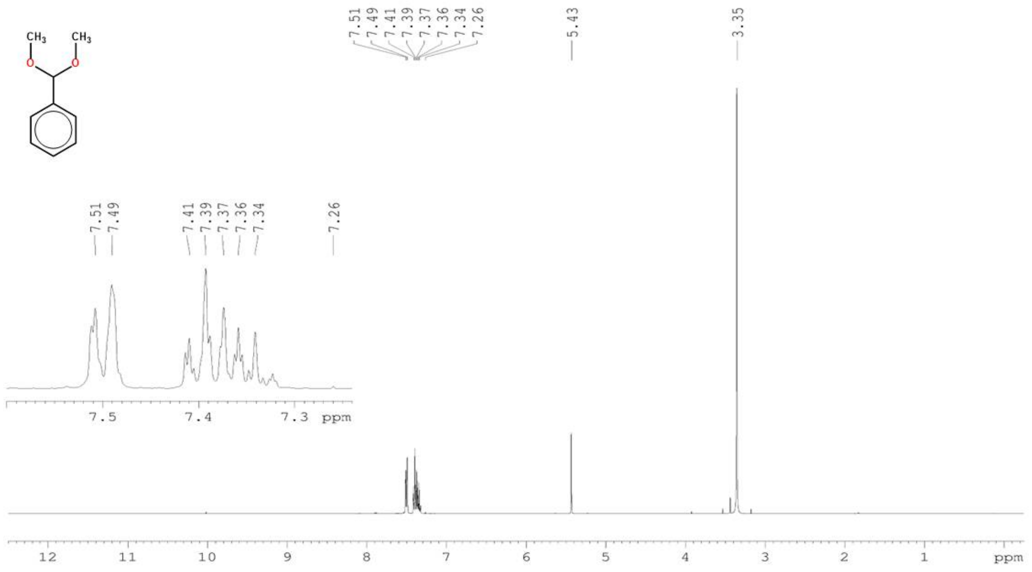

Figure S11. ^{1}H NMR (400 MHz, $CDCl_3$) spectrum of benzaldehyde dimethyl acetal (BDMA).

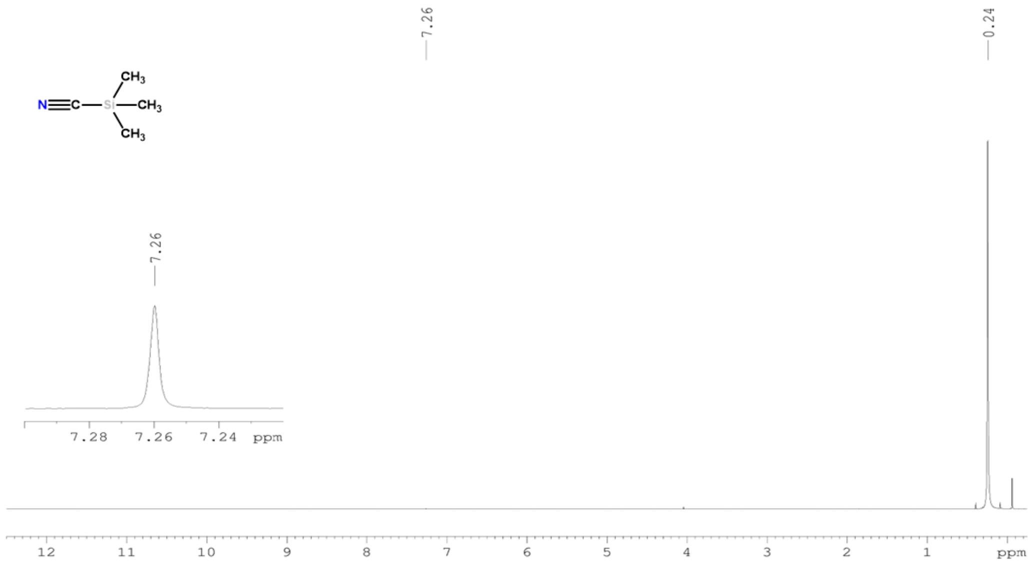

Figure S12. ^{1}H NMR (400 MHz, $CDCl_3$) spectrum of Trimethylsilyl cyanide (TMSCN).

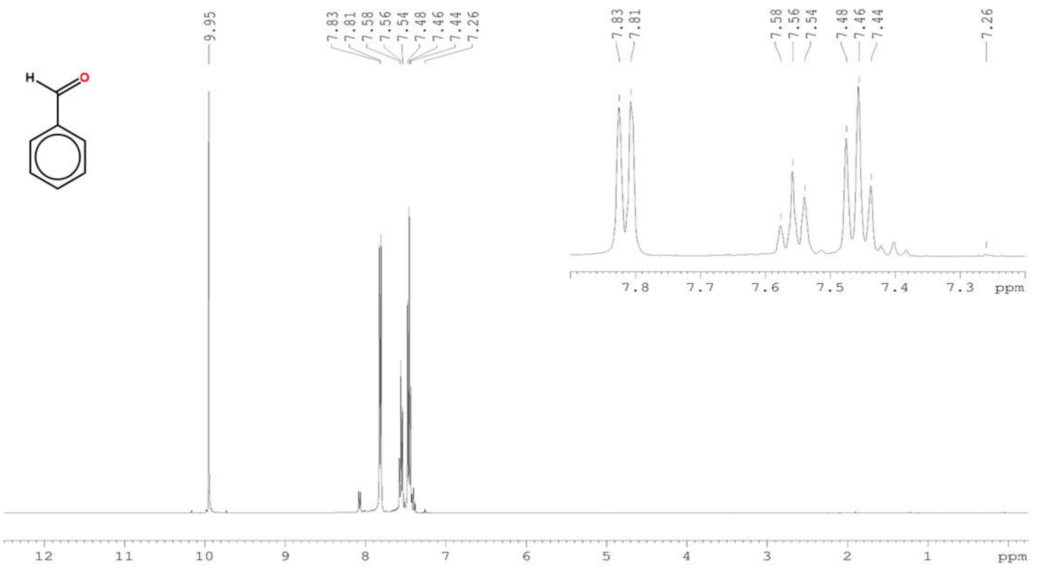

\

Figure S13. ¹H NMR (400 MHz, $CDCl_3$) spectrum of benzaldehyde (BA).

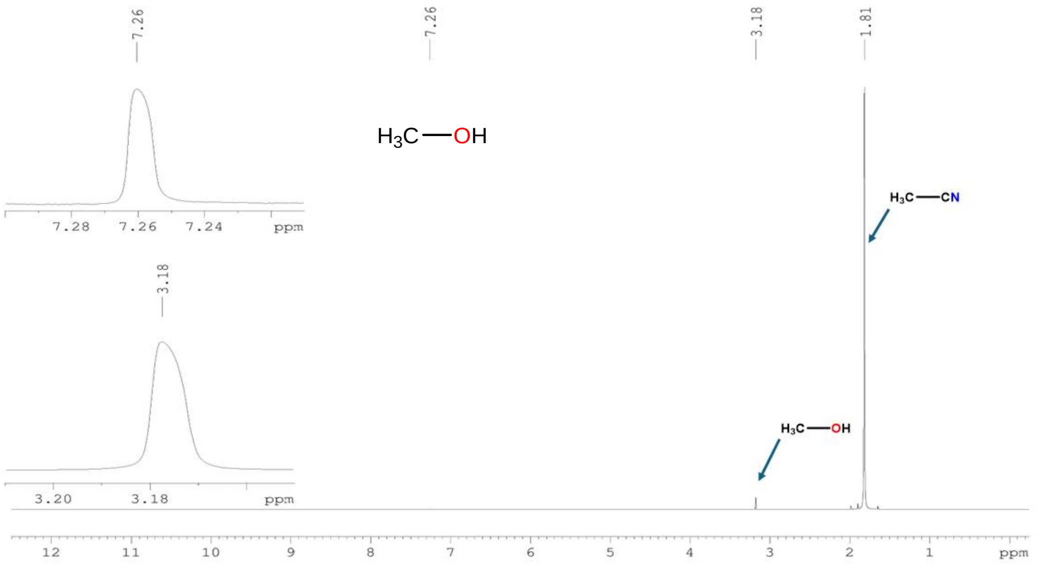

Figure S14. ¹H NMR (400 MHz, $CDCl_3$) spectrum of MeOH.

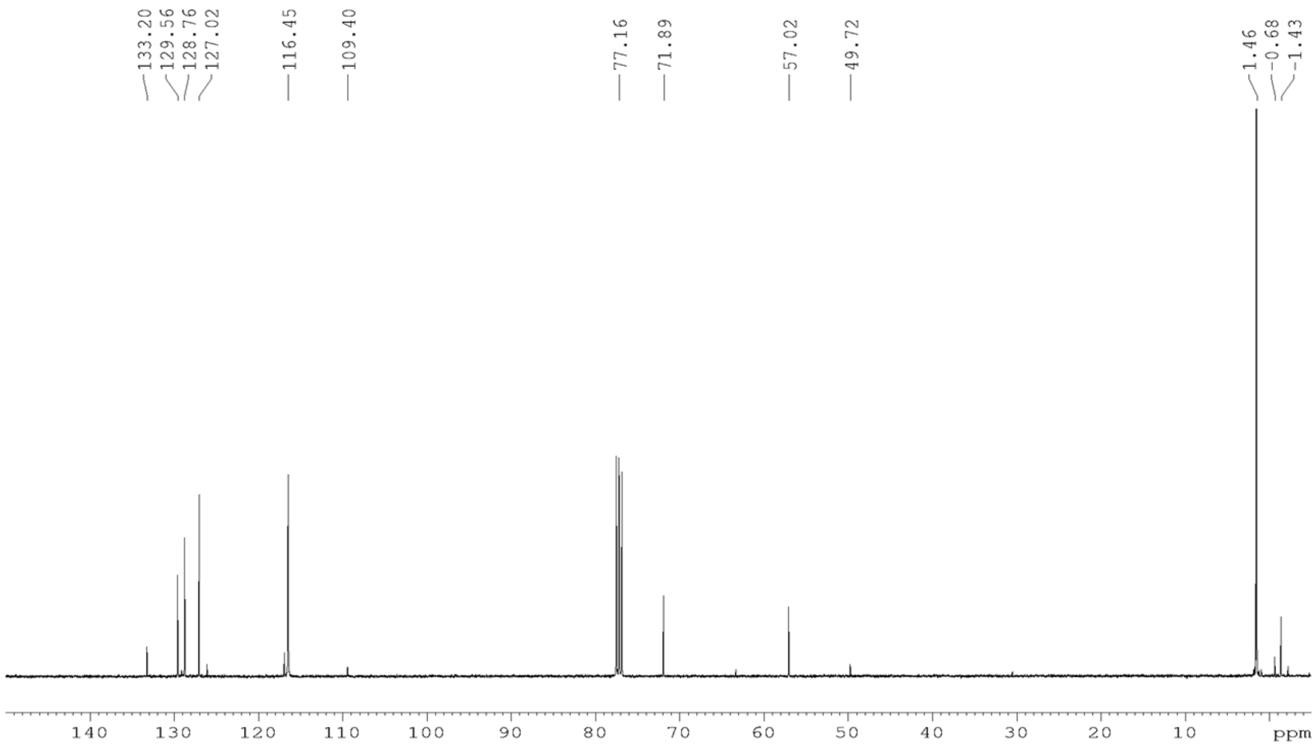

Figure S15. ^{13}C NMR (400 MHz, $CDCl_3$) spectrum of products of the reaction between BDMA and TMSCN (1 : 2) catalyzed by $SnCl_4{\bullet}5H_2O$ (**^{13}C NMR (400 MHz, $CDCl_3$) δ 133.20** (Ar-C; PhCH(OMe)(CN)), **129.56**, **128.76**, **127.02** (Ar-CH; PhCH(OMe)(CN)), **116.45** (overlapping CN signals from PhCH(OMe)(CN), CH_3CN, and TMSCN), **109.40** (HCN), **71.89** [CH; PhCH(OMe)(CN)], **57.02** (OCH_3; PhCH(OMe)(CN)), **49.72** (CH_3OH), **1.46** (CH_3CN), **0.68** [$Si(CH_3)_3OH$], **−1.43** [$Si(CH_3)_3CN$]).
